# Post-Harvest Prevention of Fusariotoxin Contamination of Agricultural Products by Irreversible Microbial Biotransformation: Current Status and Prospects

**DOI:** 10.3390/biotech12020032

**Published:** 2023-05-05

**Authors:** Natalia V. Statsyuk, Sophya B. Popletaeva, Larisa A. Shcherbakova

**Affiliations:** All-Russian Research Institute of Phytopathology, 143050 Bolshie Vyazemy, Russialarisavniif@yahoo.com (L.A.S.)

**Keywords:** fusariotoxins, irreversible microbial biotransformation, enzymatic degradation, zearalenone, deoxynivalenol, fumonisin B1

## Abstract

Biological degradation of mycotoxins is a promising environmentally-friendly alternative to chemical and physical detoxification methods. To date, a lot of microorganisms able to degrade them have been described; however, the number of studies determining degradation mechanisms and irreversibility of transformation, identifying resulting metabolites, and evaluating in vivo efficiency and safety of such biodegradation is significantly lower. At the same time, these data are crucial for the evaluation of the potential of the practical application of such microorganisms as mycotoxin-decontaminating agents or sources of mycotoxin-degrading enzymes. To date, there are no published reviews, which would be focused only on mycotoxin-degrading microorganisms with the proved irreversible transformation of these compounds into less toxic compounds. In this review, the existing information about microorganisms able to efficiently transform the three most common fusariotoxins (zearalenone, deoxinyvalenol, and fumonisin B1) is presented with allowance for the data on the corresponding irreversible transformation pathways, produced metabolites, and/or toxicity reduction. The recent data on the enzymes responsible for the irreversible transformation of these fusariotoxins are also presented, and the promising future trends in the studies in this area are discussed.

## 1. Introduction

Mycotoxins are secondary metabolites of a number of soil and plant pathogenic fungi, causing toxic responses in humans and animals. Microorganisms producing mycotoxins include a number of genera, among which *Aspergillus*, *Fusarium*, and *Penicillium* are of the most concern on a global scale [1].

*Fusarium* fungi are among the most harmful plant pathogens infesting cereal crops in temperate climatic zones [2]. These fungi cause such economically important cereal diseases as Fusarium head blight, Fusarium ear rot, and are also responsible for root and foot rots causing pre-/post-emergence death of cereal seedlings [3] thus significantly reducing crop yield and grain quality. Some fungi of this genus produce various fusariotoxins including zearalenone (ZEN), deoxynivalenol (DON), and fumonisins. According to a recent report by BIOMIN, a referenced company in the field of mycotoxins [4], these mycotoxins are the most prevalent on cereals and cereal products in the world (Table 1); they are also among the most important and regulated mycotoxins on a global scale [5].

**Figure 1:**
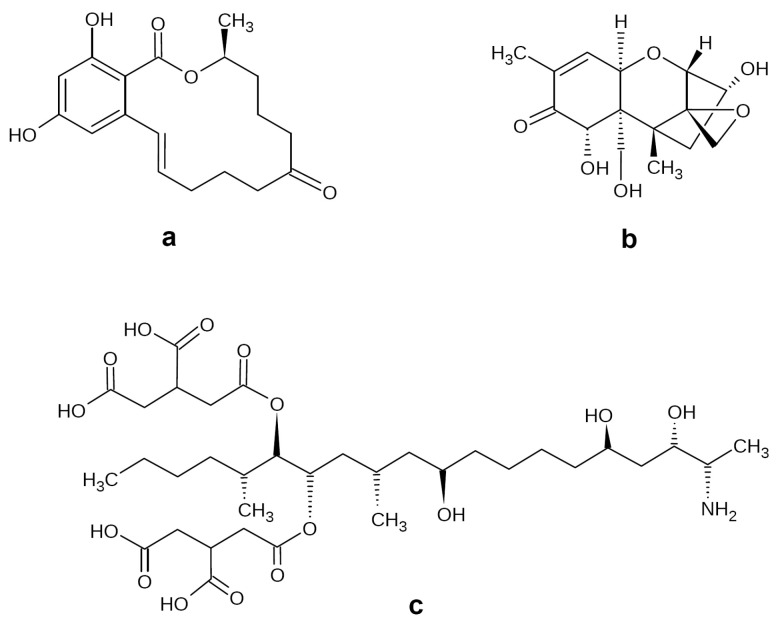
Chemical structures of (**a**) zearalenone, (**b**) deoxynivalenol, and (**c**) fumonisin B1.

ZEN is produced by a number of *Fusarium* fungi, including *F. graminearum*, *F. culmorum*, *F. cerealis*, and *F. oxysporum* [6]. This mycotoxin is synthesized via a polyketide pathway, and its structure is similar to naturally occurring estrogens (Figure 1a). Probably due to this fact, ZEN has strong estrogenic and anabolic properties, causing various reproductive disorders and related economic losses in husbandry [7]. In addition, it is characterized by immunotoxic, hepatotoxic, cytotoxic, genotoxic, and xenogenic properties [6]. Compared to DON, ZEN possesses significantly less phytotoxicity [8].

DON belongs to the trichothecene B group of mycotoxins and is classified as a tetracyclic sesquiterpene [9] (Figure 1b). The main producers of this mycotoxin are the *F. graminearum* and *F. culmorum.* DON possesses some phytotoxic effects, such as necrosis and chlorosis, and may be considered a virulence factor enhancing the phytopathogenic properties of its producers [8]. DON, which is also referred to as “vomitoxin”, causes various digestion disorders, such as nausea, diarrhea, emesis, and feed refusal in livestock; in addition, DON and related trichothecenes, such as 3-acetyl-DON, also cause allergic skin reactions, leukocytosis, and hemorrhages, as well as adverse effects on reproduction and development [10].

Fumonisins are produced primarily by *F. verticillioides* and also some other *Fusarium* fungi (*F. proliferatum*, *F. fujikuroi*, *F. nygami*, *F. oxysporum*), as well as by *Alternaria alternata* f. sp. *Lycopersici* and *Aspergillus niger* [11]. To date, 28 structurally related fumonisin analogues are known [12], among which fumonisin B1 (FB1) represents the main and the most toxic contaminant accounting for ~70–80% of the total fumonisin contamination cases [12,13]. FB1 belongs to sphingosine analogues (Figure 1c) and is synthesized via a polyketide pathway [14,15]. Feeding with FB1-contaminated forage may result in neurotoxic, hepatotoxic, and nephrotoxic effects in animals [16]. FB1 has been classified by the International Agency for Research on Cancer as a possible carcinogen for humans and may cause human esophageal cancer and neural tube defect disease [17,18]. In addition, this mycotoxin is known for some phytotoxic effects in maize, such as reduced shoot and root length and dry mass and necrotic lesions on leaves; in addition, the spraying of some other weed and crop plants with FB1 caused such effects as reduced germination, chlorosis, necrosis, black leaf lesions, stunting, wilting, defoliation, and plant death [9]. Fumonisins also gain some advantages for their producers by inhibiting the growth of their competitors and regulating their environment [19].

To avoid the negative effects of fusariotoxins on animals, humans, and crops, two basic strategies based on pre- and post-harvest activities can be implemented. The first one includes measures intended to prevent crop contamination by mycotoxin-producing fungi, such as various agronomic methods (crop rotation, tillage, sowing dates, and proper irrigation), use of resistant crop varieties, and plant protection using fungicides and biocontrol agents [3]. However, these methods are not able to provide a complete suppression of fungal development and fusariotoxin production. Agronomic methods are able to reduce mycotoxin accumulation only up to a certain level, and their efficiency strongly depends on other factors. Natural plant resistance to fungi and mycotoxins have a complex character since it is determined by many QTL (quantitative trait loci) and manifested in a different degree depending on a number of environmental factors. The use of genetically modified plants, such as *Bt* maize, intended to reduce wounding of plants by insects facilitating the further plant infection with certain *Fusarium* species, showed some reduction of the fumonisin content in the grain [20], but some studies demonstrated that the field location and weather factors provided a larger contribution to the total fumonisin content than the *Bt* trait itself [21]; moreover, use of genetically modified plants is prohibited in a number of countries. Chemical control with fungicides may become less effective with time due to mutations and the development of resistant fungal strains. Additionally, fungicidal treatments of crops, especially in low doses, may result in chemical stress in attacked fungi, causing even more active synthesis of mycotoxins as a response [22]. Application of antagonistic fungi and bacteria to reduce the incidence and level of crop contamination with *Fusarium* fungi under field conditions may have a different efficiency depending on weather conditions, as well as on the soil and microbial environment; moreover, there are some other concerns for their use including their possible effects on other microflora, the transformation of antagonistic atoxigenic strains into pathogenic and/or toxigenic ones due to horizontal gene transfer, and any health risks (allergies, etc.) [23]. There is also a shortage of commercial microbial-based products that can be used to control *Fusarium* fungi in the field along with current Fusarium head blight management strategies [24]. Last but not least, since toxigenic *Fusarium* fungi may grow as facultative parasites on plants and, at the same time, be saprotrophs, it is almost impossible to completely prevent their development on grain, sylage, and other stored products. Therefore, post-harvest prevention of the accumulation of fusariotoxins or their degradation remains very relevant.

Hand or mechanical sorting, washing, and husking of harvested grain is able to significantly reduce the mycotoxin content, but they are time- and labor-consuming. Fusariotoxins are very resistant to physical or chemical treatment. This makes some problems in the detoxification of contaminated products by physical or chemical methods, and almost all such approaches (high-temperature heating, ozonation, nixtamalization, use of arch discharge plasma, treatment with antioxidants, etc. [25,26]) have some disadvantages, such as laboriousness, expensiveness, environmental pollution, or changes in the quality and organoleptic characteristics of the treated products. This stimulates the search for new efficient and, at the same time, environmentally safe methods, for which the application would keep the quality of treated products. From this point of view, the use of microorganisms or functional enzymes able to transform mycotoxin molecules into nontoxic metabolites is one of the promising approaches for mycotoxin control that is confirmed by a large number of such studies published in recent decades.

Due to an increasing number of studies describing new strains and enzymes able to degrade mycotoxins, the landscape of this field of science is rapidly changing. Most of the studies on the biodegradation of the three above-described fusariotoxins deal with ZEN and DON, while the number of similar studies on the FB1 control is significantly lower. In recent years, a number of reviews presenting information about various microorganisms, as well as microbial products able to detoxify contaminated products, have been published [2,18,27,28,29,30,31,32,33]. However, the majority of published studies on the microbial strains able to remove mycotoxins do not include data confirming the occurred irreversible transformation of the toxin molecules into nontoxic (or at least less toxic) compounds. At the same time, the observed effects of mycotoxin removal, which is often registered via quantification of the residual mycotoxin content by high-pressure liquid chromatography (HPLC), may be determined not only by a real transformation of the toxin molecules into nontoxic or less toxic compounds but also by mycotoxin absorption by the tested microorganisms or formation of modified mycotoxins that are recovered in the course of their metabolization in the gastrointestinal tract. In this regard, information confirming the ability of the tested microorganisms to irreversibly transform mycotoxin molecules into nontoxic compounds is important since it is crucial for the evaluation of the practical value of the revealed detoxifying agents as the sources of toxin-degrading enzymes potentially suitable for practical application.

As far as the authors know, no reviews focused only on studies describing the real irreversible microbial transformation of fusariotoxins into less toxic compounds have been published. Thus, the main focus of this review will be put on studies in which the authors confirmed (or at least suggested) that the tested microorganisms were able to transform the most important fusariotoxins (ZEN, DON, and FB1) into less toxic products via the known irreversible pathways. Special attention will be also paid to the corresponding enzymes identified from such microorganisms. The authors hope this collected and analyzed information may be of practical importance for researchers working in this field of study.

## 2. Biotransformation of Zearalenone

### 2.1. Zearalenone Biotransformation Pathways

**Figure 2:**
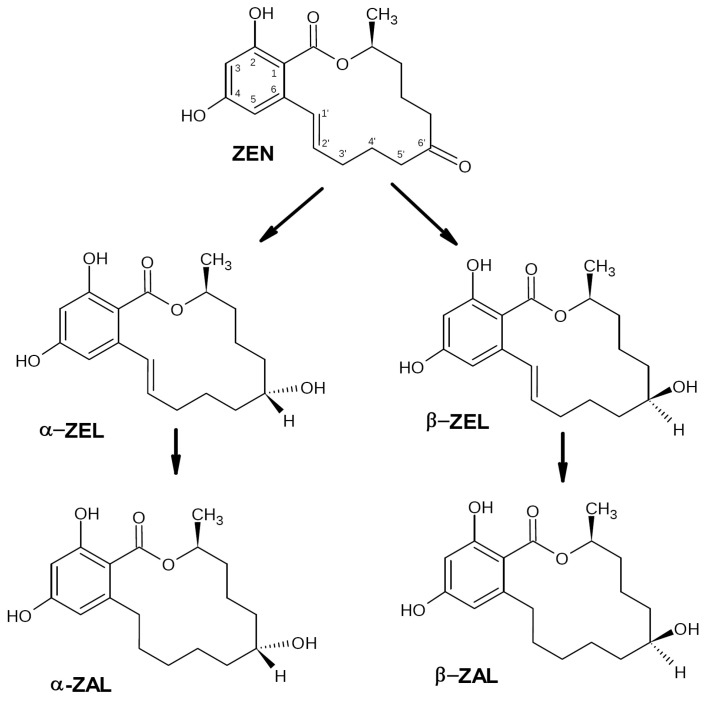
Biotransformation of zearalenone into main metabolites in mammal gastrointestinal tract. ZEN, zearalenone; α-/β-ZEL, α-/β-zearalenol; α-/β-ZAL, α-/β-zearalanol.

ZEN biotransformation in animals and humans occurs via a reduction of the C6′-ketonic carbonyl group (Figure 2) with formation of two main metabolites, α-zearalenol (α-ZEL) and β-zearalenol (β-ZEL). The further reduction of these metabolites destroys the C1′–C2′ double bond with the formation of α- and β-zearalanol (α-/β-ZAL) [34]. All these metabolites still have estrogenic activity, which is the highest for α-ZEL (even compared with ZEN) and the lowest for β-ZEL [35]. To date, a variety of microorganisms was shown to transform zearalenone into α- and β-ZEL. This ability was reported, for example, for *F. gibbosum*, some yeasts, and rumen microorganisms [36,37,38]. However, since these metabolites still keep the estrogenic activity, such biotransformation cannot be considered as detoxification.

The described pathways for microbial ZEN biotransformation relate mainly to the modification of the phenolic hydroxyl groups, cleavage of the lactone ring, and cracking of the dihydroxybenzene ring.

Oxidation of the phenolic hydroxyl groups (C2/C4-OH) with the formation of sulfate and glucoside derivatives was reported for some *Rhizopus*, *Fusarium*, and *Aspergillus* species [30]. Recently, a novel ZEN transformation mechanism through phosphorylation at the C4 position was reported for *Bacillus* sp. [39]. At the same time, mycotoxin derivatives of such type are often considered as conjugated or “modified” mycotoxins, which may release initial mycotoxins during their metabolization in mammals, thus recovering the initial toxicity level [40,41,42,43]. Therefore, this biotransformation pathway cannot be considered being effective in vivo.

**Figure 3:**
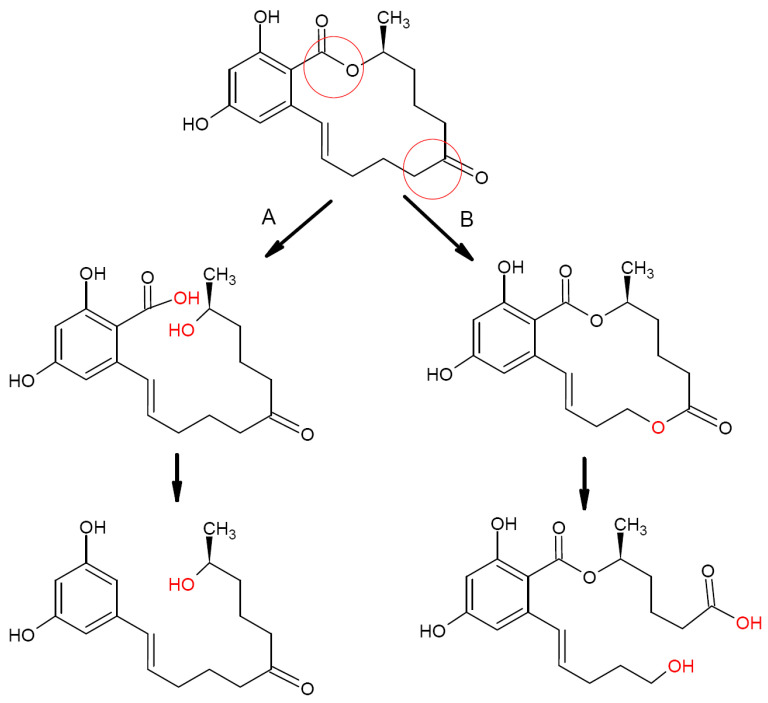
Zearalenone transformation pathways resulting in the irreversible loss of its estrogenic properties via the cleavage of the lactone ring. (A) Lactone ring cleavage. (B) Cracking of the C6′ ketone carbonyl group. The cleavage points are indicated with red. Adapted from [30].

The most efficient and safe way for ZEN biotransformation is a cleavage of the lactone ring or cracking of the C6′-ketone carbonyl group (Figure 3). In this case, the resulting derivatives lose a significant part of their estrogenic activity and become nontoxic, as shown in a number of studies. Another possible mechanism for the loss of estrogenic activity suggested in some publications is cracking the dihydroxybenzene ring [30]. Data on microorganisms providing such ZEN biotransformation pathways are summarized below.

### 2.2. Microbial Transformation of Zearalenone into Nontoxic Metabolites

To date, the number of studies confirming microbial transformation of ZEN into nontoxic (in terms of estrogenicity) products is relatively low compared to the total number of publications in this field. The reported microorganisms capable of metabolizing ZEN into nontoxic compounds include bacteria, fungi, and yeasts.

#### 2.2.1. ZEN-Transforming Bacteria

The analysis of published data on known bacteria confirmed to be able to transform ZEN into non-estrogenic products shows they belong to *Bacillus*, *Rhodococcus*, *Pseudomonas*, and *Acinetobacter* species.

***Bacillus* sp**. In Vitro studies of two *Bacillus* strains, *B. subtilis* 168 and *B. natto* CICC 24640, showed each of them was able to effectively degrade ZEN [44]. After a 24 h aerobic incubation at 30 °C in a liquid medium supplemented with ZEN (20 μg/L), the level of ZEN conversion was 81 and 100%, respectively. The performed mass spectrometry analysis did not reveal estrogen-type ZEN analogues among the resulting metabolites. According to the authors of the study, the ZEN-degrading process was accompanied with CO_2_ release indicating decarboxylation reactions and was associated with a 31–43-kDa metalloproteinase.

Screening of soil bacteria for their esterase activity with the further testing of selected strains for their ability to degrade ZEN revealed that the *B. pumilis* strain ES-21 was able (after optimization of reaction conditions) to degrade up to 95.7% ZEN added to a liquid medium to a final concentration of 17.9 μg/mL after a 24 h incubation at 40 °C [45]. The degradation product was identified as 11-(3,5-dihydroxyphenyl)-6′-hydroxy-l′- undecen-l0′-one formed via the cleavage of the lactone ring followed by decarboxylation.

The *B. velezensis* strain ANSB01E, isolated from chicken cecal content, efficiently degraded ZEN in both LB and corn meal media (95 and ~75%, respectively) after a 48 h incubation at 37 °C [46]. The genome analysis of ANSB01E revealed the presence of genes encoding peroxyredoxin and alpha/beta hydrolase. Peroxyredoxin shared 68% amino acid identity with its ZEN-degrading analogue from *Acinetobacter* sp. SM04 reported to degrade ZEN to less estrogenic products via cracking of the ketone carbonyl group [47]. The revealed alpha/beta hydrolase (GenBank accession no. QBK11187.1) had 24% identity with the ZEN-degrading lactonase Zhd101 from *Clonostachys rosea* that was able to cleave the lactone ring [48].

***Rhodococcus* sp.** Cserháti et al. (2013) screened 32 *Rhodococcus* strains for their ability to degrade several economically important mycotoxins (aflatoxin B1 (AFB1), ZEN, FB1, T2 toxin, and ochratoxin A) [49]. The tested strains were grown in LB liquid medium supplemented with 2 ppm (2 μg/mL) of a target mycotoxin for 72 h at 28 °C with the further evaluation of residual mycotoxin content by HPLC (ZEN) or ELISA (ZEN, α-ZEL, β-ZEL, α-ZAL, β-ZAL). The safety of ZEN degradation products was evaluated using the Bioluminescent Yeast Estrogen (BLYES) bioassay. Almost half of the studied strains were able to degrade ZEN. The best results were shown by the *R. pyridinivorans* strains K402, K404 and K408 (ZEN degradation by 70.1, 72.3 and 80.3%, respectively, with a complete cease of estrogenicity of degradation products). *R. ruber* N361 and R. *erythropolis* NI1 degraded ZEN by 60 and 50%, respectively, with a 40–60% reduction of estrogenicity. The earlier study reported the above-mentioned strains did not produce cytotoxic metabolites [50]. Other strains included in the study demonstrated lower ZEN-degrading efficiency. The K408 strain was also tested in vivo using an immature uterotrophic assay [51]. Prepubertal female rats were treated with feed supplemented with LB broth containing 500 mg/L ZEN that has been preliminarily incubated with or without the K408 strain. The estrogenic effect was evaluated by the uterine weight and the mRNA level changes relating to apelin, aquaporin 5, complement component 2, and calbindin-3 genes representing the major pathways affected by estromimetic compounds. “ZEN” feeding significantly increased the uterus weight in a dose dependent manner, upregulated complement component 2 and calbindin-3 expression, and decreased apelin and aquaporin 5 mRNA levels comparable to the control. In contrast, “ZEN–K408” feeding did not display any estrogenic effect either on uterine weight or on the expression of estrogen-regulated genes. Based on these results, the authors concluded the K408 strain was proved to be a very efficient biological tool for the elimination of ZEN estrogenic effects.

Several years later, the same team screened 42 *Rhodococcus* strains for the ability to degrade AFB1 and ZEN [52]. Experimental conditions included a 72 h incubation of the tested strains at 28 °C in the LB medium supplemented with 1 μg/mL of ZEN. The residual estrogenicity and cytotoxicity of ZEN degradation products were evaluated using the BLYES and BLYR bioassays, respectively. For all tested strains, no cytotoxicity of the produced ZEN metabolites was reported. Only one strain, *R. percolatus* JCM 10087T isolated from wastewater sludge, was able to degrade 95% of the ZEN with a 70% loss of estrogenicity of the degradation products. Other strains were not able to reduce the estrogenic effect of ZEN remarkably (<30%). Moreover, some of the strains belonging to *R. fascians*, *R. aetherivorans*, *R. qingshengii*, *R. cerastii*, *R. canchipurensis*, *R. triatomae*, *R. coprophilus*, and *R. cercidiphylli* species were shown to transform ZEN to even more estrogenic by-products.

Screening of soil samples reported in the study [53] revealed an *R. erythropolis* PFA D8-1 strain able to convert ZEN via the cleavage of the lactone ring with a formation of hydrolyzed ZEN (HZEN). The loss of estrogenicity in the final product was confirmed by the MCF7 bioassay, as well as by the test with an estrogen reporter yeast strain YZHB817. Later the authors isolated and identified an enzyme (ZenA) responsible for the target activity and developed and tested a commercial enzymatic preparation ZENzyme^®^ (see Section 2.2.3).

***Acinetobacter* sp**. The *Acinetobacter* sp. strain SM04 isolated from agricultural soil was able to rapidly grow on M2 medium supplemented with 0.5 mg/mL of ZEN as a sole carbon source [54]. After a 72 h incubation at 30 °C, the strain almost completely utilized ZEN since neither this toxin nor its metabolites (α-ZOL and β-ZOL) were detected. Extracellular extracts of SM04 cultures grown on M1 or nutrient broth (NB) media were tested for their ability to degrade ZEN (20 μg/mL) at 30 °C by HPLC, mass spectrometry, and the MTT cell proliferation assay with MCF-7 cells (estrogen receptor-positive human breast cancer cell line possessing a proliferative response to estrogens). The authors reported that neither ZEN nor other equally estrogenic metabolites were detected after the 12 h ZEN treatment with the “M1” extracts of SM04. The authors inferred that the ZEN transformation included oxidation and cracking of the dihydrobenzene ring with a formation of compounds containing carboxy groups. Further analysis revealed some proteins in these extracts were essential for the ZEN degradation. Later, the authors reported a successful cloning and expression of the peroxiredoxin gene possessing the ZEN-degrading activity of this strain (see Section 2.2.3) [47].

***Pseudomonas* sp.** Altalhi (2007) reported the *Pseudomonas* sp. strain ZEA-1, isolated from the corn plant rhizosphere, was capable of utilizing ZEN as the sole carbon source [55]. The strain rapidly utilized ZEN at high concentrations (>200 μg/mL) and showed prolific growth on minimal medium supplemented with 100 μg/mL ZEN. After a 12 h incubation at 30 °C on this medium, >50% of ZEN was degraded; after 72 h of incubation, no residual ZEN was detected. TLC analysis of ZEN degradation products did not reveal the presence of ZEN, α-ZOL, and β-ZOL. The toxicity of the ZEN transformation products was tested on *Artemia salina* shrimps. The LD_50_ of ZEN for *A. salina* larvae was ~50 µg/mL, while little or no toxicity was observed at 5 µg/mL. A higher ZEN concentration (100 µg/mL) was associated with the *A. salina* mortality rate of 97%. At the same time, no mortality was observed for ZEN degradation products after 24 h of incubation for all three levels of the initial ZEN concentrations. Based on these results, the author suggested less toxicity of the degradation products and the possibility of using the strain for ZEN detoxification. The authors detected a 120-kB plasmid responsible for the ZEN catabolization in ZEA-1. The plasmid transferred into an *E. coli* strain resulted in obtaining some *E. coli* colonies that were able to degrade the ZEN, while plasmid-free ZEA-1 lost its ability to utilize ZEN as a sole carbon source.

#### 2.2.2. ZEN-Transforming Fungi and Yeasts

The existing publications on the fungi and yeasts with the proven ability to safely degrade ZEN into non-toxic products include the studies of *Clonostachys* (*Gliocladeum*), *Aspergillus*, and *Trichosporon* species.

***Clonostachys rosea* (=*Gliocladeum roseum*)**. The first report on the ability of *C. rosea* to degrade ZEN was published in 1988. A 72 h incubation of *C. rosea* NRRL1829 at 27 °C in a liquid medium supplemented with 250 μg/mL ZEN resulted in a 80–90% degradation of the toxin with a formation of a 1:1 mix of two isomers, 1-(3,5-dihydroxypheny1)-10′-hydroxy-1-undecen-6′-one and 1-(3,5-dihydroxyphenyl)-6′-hydroxy-l-undecen-10′-one presumably produced by the cleavage of the lactone ring with the subsequent spontaneous decarboxylation of the intermediate compound (similar to Figure 3A) [56]. According to the authors, both observed metabolites did not inhibit the estradiol binding to the estrogen receptors, i.e., they did not possess estrogenic activity.

Later the screening of a number of soil and plant microorganisms for their capability to convert ZEN to nontoxic compounds revealed a *C. rosea* strain IFO 7063 able to degrade ZEN (100 μg/mL) to a nonestrogenic product after a 96 h incubation at 28 °C [57]. The structure of the resulting cleavage product was determined by nuclear magnetic resonance (NMR) and mass spectrometry (MS) analysis, while its estrogenic properties were assessed using MCF-7 cell culture, in which growth is stimulated by estrogen and estrogen-like compounds. The cleavage product was not able to stimulate cell growth even at a 1000 times higher concentration than that of ZEN, i.e., it did not show any estrogenic activity. It was identified as 1-(3,5-dihydroxyphenyl)-10′-hydroxy-1′E-undecene-6′-one, i.e., one of the isomers described in the study [56]. Further studies of the strain allowed the authors to identify the lactonase responsible for ZEN transformation (see Section 2.2.3).

Recently it was also shown that extracellular metabolites of the *C. rosea* GRZ7 strain, which were capable of degrading AFB1 [58], possessed ZEN-catabolizing activity [59]. After a 72 h incubation of GRZ7 in the liquid medium supplemented with 0.5 μg/mL of ZEN, the toxin was degraded by 68%. Further study allowed the authors to determine a gene encoding lactonohydrolase, similar to other lactonohydrolases from *C. rosea* possessing the proven ZEN-detoxifying ability (see Section 2.2.3).

***Aspergillus niger***. Sun et al. (2014) examined spores, mycelium, and culture filtrate of the *A. niger* strain FS10 isolated from Chinese fermented soybean for their ability to degrade ZEN in vitro [60]. After a 48 h incubation at 30 °C in a potato dextrose broth (PDB) medium supplemented with ZEN (2 μg/mL), FS10 suspension could remove 89.56% of the toxin. Further study showed no ZEN removal effects for spores; in the case of mycelium, the authors concluded that the ZEN removal (43.1%) was determined by binding of the toxin rather than its transformation. In the case of culture filtrate (68.16% ZEN removal), the authors made additional experiments to conclude the effect is provided by extracellular enzymes (presumably metal-containing oxidases). A liquid chromatography-mass spectrometry (LC-MS) analysis of the degradation products revealed two intermediates, ZEN-A and ZEN-B, differing from ZEN with a probably cleaved benzene ring in the case of the last one. These degradation products were reported to be less toxic using a rat model with the liver atrophy level and liver/kidney cell histology as toxicity indicators.

However, the recent study using an ultra-high performance liquid chromatography with quadrupole time-of-flight mass spectrometry (UPLC-Q-TOF-MS) analysis of the ZEN degradation products produced by this strain reported it degrades ZEN into two products, ZEN-4-sulfate (main product) and (E)-ZEN (ZEN isomer, a minor product), i.e., no cracking of the dihydrobenzene ring occurred [61]. Compared to ZEN, ZEN-4-sulfate was significantly less toxic, while the toxicity of (E)-ZEN was not determined. The earlier bioassay studies also showed that the *Aspergillus* section *Nigri* isolates were able to transform ZEN into ZEN-4-sulfate which was characterized by less estrogenic toxicity [62]. Though sulfonation was known to be a classic detoxification pathway in mammals facilitating the excretion of xenobiotic compounds via kidneys and bile [63], ZEN-4-sulfate ingestion by rats resulted in a partial recovery of the estrogenic properties of ZEN that was revealed by the rat uterus enlargement bioassay [64]; the authors explained it by the susceptibility of this compound to acid hydrolysis occurring in the gastrointestinal tract and resulting in the release of a recovered ZEN. Thus, ZEN degradation by *A. niger* cannot be considered as irreversible.

***Trichosporon mycotoxinivorans***. Vekiru et al. (2010) reported the ability of *T. mycotoxinivorans*, a basidiomycete yeast used as a microbial feed additive, to degrade ZEN [65]. After a 48 h incubation of the yeast at 37 °C in a saline medium supplemented with 10 μg/mL ZEN, 95% of the toxin was degraded via the opening of the C6-ketone carbonyl group with formation of the main metabolite, ZOM-1, shown to be stable for at least several days. ZOM-1 was identified by the liquid chromatography-tandem mass spectrometry (LC-MS/MS), LC-diode array detector (LC-DAD), time-of-flight mass spectrometry (TOF MS), and NMR analyses as (5S)-5-({2,4-dihydroxy-6-[(1E)-5-hydroxypent-1-en-1-yl]benzoyl}oxy)hexanoic acid characterized by the cleavage of the lactone ring via cracking of the C6′ carbonyl group (see Figure 3B). ZOM-1 did not show estrogenic activity in a sensitive yeast bioassay even at a concentration 1000-fold higher than that of ZEN and did not interact with the human estrogen receptor in an in vitro competitive binding assay. Based on the obtained results and the fact that *T. mycotoxinivorans* can be fermented, concentrated, freeze-dried, and stabilized without losing its target activity, the authors concluded the use of this yeast as a feed additive for mycotoxin detoxification looks quite promising; however, enzymes involved in this two-step degradation pathway remained unclear, though the authors suggested they may include a Bayer-Villiger oxydase (BVMO) and carboxylesterase. A recent whole-genome sequencing of this species and further bioinformatic analysis using a genome-scaled prediction of substrate-specific enzyme (GPSE) revealed a C1_1820 protein, a putative BVMO enzyme most likely oxidizing ZEA to ZOM via insertion of an oxygen atom between C5′ and C6′ atoms (1st step) [66]. For the next step (breaking the ester bond at C6′ by carboxylesterase to produce ZOM-1), this in silico analysis revealed three putative carboxylesterase enzymes, C2_44, C3_70, and C5_1043.

#### 2.2.3. ZEN-Transforming Microbial Enzymes

In the Section 2.2.1 and Section 2.2.2, we reviewed a number of bacteria and fungi capable of irreversibly transforming ZEA into less toxic metabolites (summarized in Table 2). 

At the same time, authors of these studies did not always identify microbial enzymes responsible for such biotransformation. To date, the known microbial ZEN-transforming enzymes represent mainly fungal lactonases cleaving the lactone ring associated with ZEN toxicity; there are also some other enzymes of other types possessing similar activity.

**Lactonases**. The first reported lactonase possessing ZEN-degrading activity was Zhd101 lactonase from *Clonostachys rosea* IFO7063 [48]. The resulting product was identified by gas chromatography–mass spectrometry (GC-MS), NMR, and MS analyses as 1-(3,5-dihydroxyphenyl)-10’-hydroxy-1’-undecen-6’-one. However, the optimal pH of Zhd101 was 9–10 and that limited its possible application, so the gene encoding this enzyme was actively used to modify a number of organisms, such as *E. coli* and *Saccharomyces cerevisiae* [67] as well as in *Pichia pastoris* [68] and *Lactobacillus reiteri* [69]. A number of studies were carried out with other ZENG [70], Zhd795 [71], and ZEN-jjm [72] proteins highly homologous to Zhd101 and originating from different *C. rosea* strains. Proteins similar to Zhd101 were also revealed in other fungal species, such as *Phialophora americana* (ZHD607), *Cladophialophora bantiana* (CLA), *Exophiala aquamarina* (EXO), and *Trichoderma aggressivum* (TRI) [73,74].

Though the majority of described ZEN-degrading enzymes were tested using model solutions, the authors of some studies also tested their enzymes on various food and feed products. The Zlhy-6 enzyme from the *C. rosea* strain 31,535 characterized by a 98.3% identity with Zhd101 was shown to efficiently (96.3%) degrade the ZEN in the corn oil [75]. A ZEN-specific lactone hydrolase from the *C. rosea* strain GRZ7, expressed in a *Penicillium canescens* strain PCA-10 as a recombinant enzyme (PR-ZHD), was shown to completely decontaminate flour samples prepared from ZEN-polluted grain after an overnight exposure [76]. ZEN-degrading lactonase Zhd518 from *Rhinocladiella mackenziei* expressed in *E. coli* or *Lactobacillus reuteri* completely hydrolyzed ZEN in artificially infected corn grain and flour prepared from this grain [77]. ZEN lactonase from *Neurospora crassa* (ZENC) expressed in *P. pastoris* was successfully tested on three different kinds of animal feed (distiller dried grains, maize by-products, and corn bran) and showed a good target activity that varied from 70.9 to 94.7% [78]. Finally, ZenA, a zearalenone hydrolase derived from *Rhodococcus erythropolis* PFA D8-1, was shown to possess a higher substrate affinity and catalytic rate than ZHD101, and successfully cloned in *E. coli.* [53]. The enzyme manufactured by BIOMIN Holding GmbH (Getzersdorf, Austria) with the commercial name Zenzyme^®^ was successfully tested on cows, pigs, chickens, and rainbow trout [79,80].

**Peroxidases**. In the case of *Acinetobacter* sp. SM04, a peroxiredoxin (*Prx*) gene was revealed, cloned, and expressed in *E. coli* [81]. The resulting recombinant enzyme belonged to thio-redoxin peroxidases and was able to efficiently degrade ZEN in the presence of H_2_O_2_ probably via the cracking of the dihydrobenzene ring. Interestingly, the optimum temperature for its activity was 70 °C. Further experiments with ZEN-contaminated and crushed corn grain showed Prx degraded ~90% of the mycotoxin in 6 h at 30 °C in the presence of 0.09% H_2_O_2_.

The authors of the above-mentioned study recently reported about a novel ZEN-detoxifying enzyme (Ase) from the strain SM04 identified as an oxygen-utilizing protease containing a number of heme prosthetic groups [48]. The enzyme was able to degrade 88.4% of ZEN (20 μg/mL) within 8 h at 50 °C and pH 9. Though the structural identification of the resulting ZEN metabolites is still unknown, their estrogenic activity is lower than that of ZEN. Compared to Prx, Ase did not require H_2_O_2_ for successful ZEN degradation, but possessed a high activity under alkaline conditions that may complicate its application in the gastrointestinal environment.

**Other peroxidases**. ZEN degradation activity was also reported for dye-decolorizing peroxidase, a ligninolytic enzyme from *Streptomyces thermocarboxydus* combining catalytic properties of manganese peroxidase and laccase (two types of enzymes able to degrade AFB1). The target activity of a recombinant *St*DyP enzyme in the presence of a 1-hydroxybenzotriazole (1-HBT) as a mediator resulted in complete ZEN degradation (0.003 mM) after a 48 h reaction at pH 5 and 30 °C [82]. The LC-MS study of degradation products identified them as 15-OH-ZEN and 13-OH-ZEN-quinone, for which the toxicity might be significantly reduced compared to ZEN; according to the study [83], 15-OH-ZEN is 98% less estrogenic than ZEN. At the same time, there are still no data confirming the stability and irreversibility of such ZEN transformation.

Note that non-microbial organisms, such as white-rot fungi and plants, also produce some peroxidases capable of the transformation of ZEN and some other mycotoxins, such as DON, FB1, and AFB1, in the presence of various mediators (see, for example, Refs. [84,85]). However, these sources of enzymes are beyond the framework of this review, so they were not included in the consideration.

**Laccases**. Like peroxidases, many laccases are capable of degrading several mycotoxins and require the presence of mediators to efficiently degrade them. ZEN-degrading activity was revealed in some bacteria. For example, CotA laccase from *Bacillus licheniformis* was able to degrade ZEN in both model solutions and contaminated corn meal [86]. In the last case, the free and immobilized enzyme degraded 70 and 90% of ZEN (10 μg/g), respectively, after a 12 h incubation at 37 °C, but the optimum temperature for this activity was 75 °C, i.e., the enzyme was thermostable. The estrogenicity reduction in degradation products was confirmed by a MCF-7 bioassay, though the products were not exactly identified. Note that the results obtained for the enzyme immobilized on chitosan particles may result in the development of a commercial biocatalyst for ZEN detoxification. One should also mention that Novozyme patented the process of ZEN degradation by laccase from *Streptomyces coelicolor* applied together with phenotiazin-10-propionic acid or methylsyringate as redox mediators [87].

In addition, some laccases from white-rot fungi (*Pleurotus eryngii*, *P. pulmonarius*, and *Trametes versicolor*) were also reported to degrade ZEN [88,89,90]. However, since their sources are not microbial, we did not include them in the consideration.

**Coenzyme A thioesterases**. Transcriptomic analysis of *Bacillus amyloliquefaciens* H6 possessing ZEN-degrading activity revealed a BAMF_RS30125 gene associated with this activity [91]. The corresponding recombinant protein ZTE138 reduced the ZEN content in the medium by 59.79% after a 72 h incubation at 37 °C and also almost thrice reduced ZEN-promoted proliferation in MCF-7 cells. Sequence alignment of ZTE138 showed it belongs to the coenzyme A thioesterases and, therefore, may cleave the lactone ring responsible for the ZEN toxicity; however, a more thorough study of ZEN metabolites is required. 

**Other enzymatic ZEN-degrading activity**. A 120-kb pZEA-1 plasmid, from the *Pseudomonas* sp. strain ZEA-1 responsible for the ZEN catabolization by this strain [56], was used for transformation of *E. coli* and the further localization of ZEN degradation genes [92]. The revealed 8.6-kbp fragment with the target activity was subcloned resulting in a 5.5-kbp Pst1-Kpn1 fragment containing gene(s) responsible for ZEN degradation. However, no further reports on the identification of the encoded enzyme(s) were found.

## 3. Biotransformation of Deoxynivalenol

### 3.1. Deoxynivalenol Biotransformation Pathways

DON metabolization within the gastrointestinal tract of humans and animals occurs via de-epoxidation, glucuronidation, and sulfation or sulfonation with a formation of deepoxydeoxynivalenol (DOM-1), DON-3/DON-15-glucuronide (DON-GlcA), DON-10-sulfonate (DONS), and DON-3- or DON-15-sulfates (DON-3-S/DON-15-S), respectively. De-epoxidation, which is provided mainly by the gut microbiota and glucuronidation, and is carried out by endogenous enzymes in the liver, represents the common and best-studied pathways for the majority of animals [93]. Sulfonation and sulfation pathways are typical for rats and poultry, respectively [93]. DOM-1 is characterized by a significantly lower toxicity than DON; the loss of the C12–C13 epoxy ring resulted in a 55-fold reduction of the LD_50_ value compared to DON [94]. However, DOM-1 is rarely detected in humans [94].

**Figure 4:**
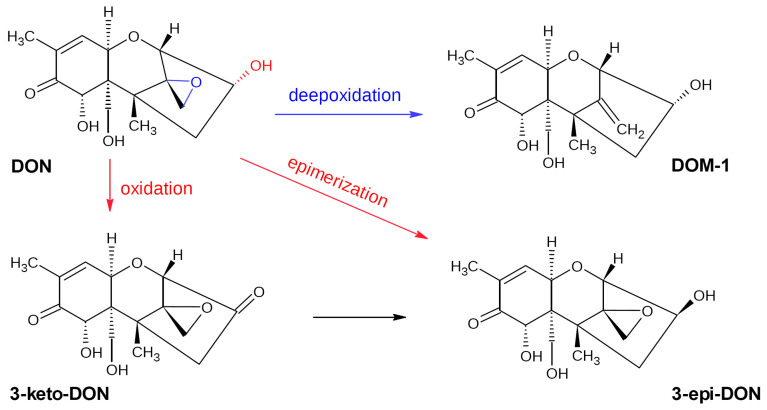
Deoxynivalenol biodegradation pathways resulting in a formation of less toxic products.

Unlike ZEN, DON is poorly eliminated by binders and absorbants due to its low polarity [95]. Therefore, the possibility of transform it into less toxic compounds is of great practical value. To date, the reported pathways for the microbial transformation of DON include de-epoxidation, epimerization, oxidation, glycosylation, glutathionylation, hydroxylation, and isomerization [96]. The first three modifications provide the safest DON metabolites (DOM-1, 3-epi-DON, and 3-keto-DON, respectively; Figure 4) [97,98]. There are a number of studies dealing with the microbial transformation of DON, though some of them just describe DON removal by mixed microbial cultures from the gastrointestinal tract of humans or animals and do not determine microbial strains possessing the target activity. Here, we review only papers, which include the description of isolated and identified bacterial or fungal species responsible for the observed effect.

### 3.2. DON-Transforming Microorganisms

Since trichothecenes are toxic for eukaryotic cells, fungi producing these mycotoxins as well as those sharing the same ecological niches with mycotoxin producers have developed some mechanisms protecting them from the harmful effects of these compounds including DON [99]. These mechanisms include acetylation of the C3 carbon by trichothecene 3-O-acetyltransferase with the formation of 3-Ac-DON revealed in some *Fusarium* fungi [100], DON hydrolyzing into conjugated DON revealed in some strains of filamentous fungi (*Alternaria alternata*, *Rhizopus microsporus*, *Aspergillus oryzae*, and *Aspergillus tubingensis*) [101,102], and DON glycosylation by some *Trichoderma* strains [103]. However, these ways of DON transformation are reversible, i.e., cannot be considered as suitable for their potential application in the food and feed industry.

To date, all reports about microorganisms capable of irreversibly transforming DON into less toxic metabolites describe various bacterial species. All reported bacteria with the confirmed ability for DON transformation can be divided into two groups. The first one includes anaerobic bacteria isolated from the gastrointestinal tracts of animals (*Bacillus* sp., *Eggerthella* sp., *Slackia* sp., *Eubacterium* sp., and *Clostridium* sp.), while the second group consists of aerobic bacteria isolated from agricultural soils (*Agrobacterium–Rhizobium* group, *Devosia* sp., *Nocardioides* sp., *Desulfitobacterium* sp*., Paradevosia shaoguanensis, Bacillus licheniformis,* and *Pelagibacterium halotolerans*). The described pathways for DON transformation occurring in bacteria include de-epoxidation for anaerobic species and epimerization/oxidation for aerobic species, though some exceptions exist. For example, Islam et al. (2011) described a mixed bacterial culture isolated from soil and consisted of six genera (*Serratia*, *Clostridium*, *Citrobacter*, *Enterococcus*, *Stenotrophomonas*, and *Streptomyces*), which was capable of de-epoxifying DON to deDON under both aerobic and anaerobic conditions [104]. The same properties were also reported for *Desulfitobacterium* sp. PGC-3-9 isolated from agricultural soils; recently, *Citrobacter freundii* also belonging to soil bacteria was shown to convert DON into DOM-1, and the authors did not mention about anaerobic conditions of its cultivation (see Section 3.2.2 for details).

#### 3.2.1. Anaerobic Bacteria

***Bacillus* sp**. Yu et al. (2010) evaluated the ability of microbial cultures from chicken intestines to transform DON [105]. The authors reported the LS100 isolate was capable of almost completely transforming DON (200 μg/mL) via a de-epoxidation pathway after a 72 h incubation at 37 °C in a liquid L10 medium. The resulting metabolites were identified by LC-MS analysis. Further study of the strain identified as putative *Bacillus arbutinivorans* found it was able to completely transform DON into DOM-1 at a concentration of 100 μg/mL within 24 h incubation at 37 °C under anaerobic conditions [106]. The isolate kept the target activity even after 10× of subculturing. It was also able to completely detoxify moldy corn containing 124 μg/g DON via its transformation into DOM-1. Feeding of pigs with moldy or DON-free corn treated or untreated with LS100 showed that the feed intake, weight gain, and feed efficiency for the “DON-LS100” and “LS100” variants did not differ from the control and exceeded the values of these parameters in the “DON” variant by 45, 82, and 32% on average.

***Raoultibacter* (=*Eggerthella*) sp**. A novel DON-transforming bacterial strain, *Raoultibacter* sp. DII-9, was isolated from chicken intestines using DON as a medium component [107]. The strain was capable of completely transforming DON (100 μg/mL) into DOM-1 via a de-epoxidation pathway after a 24 h incubation at 37 °C under anaerobic conditions. The transformation was confirmed by the HPLC and MS analysis of the resulting products. The strain was also able to de-epoxidize several other trichothecene toxins and was characterized by a broad temperature and pH tolerance. Further study showed that only live DII-9 bacteria, but not dead cells or protein extracts, possessed de-epoxidation activity to DON suggesting the DON transformation was a complex process. Genome sequencing of DII-9 did not provide clear results concerning genes encoding enzymes potentially able to degrade DON.

***Slackia* sp**. In 2020, a novel DON-transforming bacteria, *Slackia* sp., was isolated from chicken intestines using DON-containing broth [108]. The strain D-G6 completely transformed DON at the concentration of 25 μg/mL within 24 h at 37 °C under anaerobic conditions; it was also capable of transforming >90% of the DON at a concentration of 100 μg/mL within 48 h. The DON to DOM-1 transformation was confirmed by an HPLC analysis. Though the genome analysis did not allow the authors to determine genes responsible for the target activity, it made it possible to suggest that de-epoxidation may be a redox process involving flavin adenine dinucleotide (FAD), nicotinamide adenine dinucleotide phosphate (NADPH), and/or NADH.

***Coriobacterium* sp**. A microbial strain isolated from bovine rumen fluid was shown to completely convert DON (100 ppm) to DOM-1 in a modified M10 medium when cultivated under anaerobic conditions at 37 °C; identification of degradation products was performed by reversed-phase high-performance liquid chromatography (RP-HPLC) and thin-layer chromatography (TLC) [109]. The strain determined as *Eubacterium* sp. (Biomin^®^ BBSH 797), was shown to degrade some other trichothecene mycotoxins structurally related to DON and was incorporated into commercial microbial feed additives developed by the BIOMIN company for degradation of trichothecenes and authorized by EU [110]. The toxicity of the degradation products was evaluated by a lymphocyte proliferation assay (LPA) [111]. Later, the strain was re-classified as *Coriobacteriaceum* DSM 11,798 [112]. Though the earlier studies showed a positive effect of Biomin^®^ BBSH 797 on growth parameters of piglets fed by DON-contaminated food, the authors of the last study reported this additive did not provide any significant influence on growth and blood parameters as well as DON metabolism in growing pigs [112]. The authors suggested the lack of activity of DSM 11,798 may be caused by the feed pelleting process or stabilizing matrix.

***Clostridium* sp.** The *Clostridium* sp. WJ06 strain isolated from the chicken intestinal tract was shown to convert DON (20 ppm) into DOM-1 with >90% efficiency in the course of a 72 h incubation in the L10 medium under anaerobic conditions at 37 °C [113]. The mycotoxin degradation was controlled by the LC-MS/MS method. Feeding of pigs with the DON-contaminated diet supplied by WJ06 showed the toxic effects of DON on the growth performance, intestinal morphology, relative organ weight, and intestinal flora were relieved after the addition of this strain.

#### 3.2.2. Aerobic Bacteria

***Agrobacterium–Rhizobium* group**. The E3-39 isolate obtained from soil and belonging to the *Agrobacterium–Rhizobium* group was shown to almost completely transform DON (200 μg/mL) into 3-keto-DON (70%) and two other minor metabolites in the course of a 24 h incubation in BYE medium [114]. The transformation product was identified by NMR. The decreased immunosuppressive toxicity of 3-keto-DON (>90%) was confirmed by a bioassay based on the mitogen-induced and mitogen-free proliferation of mouse spleen lymphocytes. E3-39 was also capable of transforming 3-acetyl-DON (3-ADON), one of the natural DON derivatives also possessing a significant toxicity; the transformation rate reached ~75%. However, no follow-up publications or patents on the strain have appeared since that time, so probably the strain or its activity was lost [31].

***Devosia* sp**. To date, there are a number of publications describing the DON-degrading properties of Gram-negative bacteria from the genus *Devosia*. He et al. (2016) reported the *D. mutans* strain 17-2-E-8 isolated from agricultural soil and capable of transforming DON to 3-epi-DON [115]. DON transformation products were identified by HPLC. At the initial DON concentration of 100 μg/mL, the DON transformation efficiency of 17-2-E-8 after a 72 h incubation in the CMB medium at 28 °C reached ~95%. The DON epimerization process was later shown to be a two-step enzymatic process that occurred via the formation of 3-keto-DON intermediates [116].

*D. insulae* A16 isolated from a wheat field soil was shown to efficiently transform DON and its derivatives, such as 3-ADON and 15-ADON [117]. After 48 h incubation in MSM medium at 35 °C, the strain degraded 88% of the initial DON (20 μg/mL). The main degradation product was 3-keto-DON produced via oxidation of the C3 hydroxyl group. In the case of both 3-ADON and 15-ADON, a preliminary deacetylation stage was observed.

*Devosia* A6-243A strain was isolated from a mixed culture of this bacterium with *Pseudomonas* sp. B6-24 was earlier shown to be capable of biotransforming DON into 3-epi-DON. The strain was shown to lose a pyrroloquinoline quinone (PQQ) synthesis gene that limited its DON-transforming ability, while B6-24 played the role of providing PQQ [118]. In the presence of exogenous PQQ (15–30 μM), A6-243 completely transformed 100 μg/mL of DON within 48 h into 3-epi-DON and also exhibited the ability to transform 3-keto-DON into 3-epi-DON. The optimal conditions for the biodegradation process included temperature of 16–37 °C and pH 7.0–10. The authors also showed that the strain was able to completely remove DON (6.7 μg/g) from DON-contaminated wheat. Based on the obtained results, the authors proposed the use of PQQ to screen bacteria capable of transforming DON.

Another *Devosia* strain D6-9 isolated from soil demonstrated extremely high activity towards DON. Under in vitro conditions, it was able to completely degrade 500 μg/mL DON within 2 h with the rate of 2.5 μg/min/10^8^ cells [119]. A time-dependent assay of DON and its metabolites showed 3-keto-DON to be the first product of transformation; then a matched accumulation of 3-epi-DON was observed in parallel with the disappearance of 3-keto-DON, so 60 min after inoculation, 3-epi-DON was the predominant product. A high target activity of the strain was also confirmed on flour prepared from wheat grain infected with PHB pathogens: the strain completely transformed DON after a 3 h incubation at 28 °C.

Screening of soil and wheat leaf microbiota in the National Institute for Agro-Environmental Sciences (Tsukuba, Japan) revealed 13 strains able to degrade DON (100 μg/mL) to concentrations below the limit of detection (0.5 μg/mL) after a 24 h incubation in the mineral salts medium (MSM) [120]. Four of these strains belonged to *Devosia* sp. A HPLC analysis of degradation products showed these strains generated 3-epi-DON with two other undetermined metabolites.

***Nocardioides* sp**. Nine of the DON-degrading strains revealed in the above-mentioned study [120] represented the Gram-positive genus *Nocardioides*. These strains demonstrated an enhanced growth in the DON-containing MSM medium compared to the DON-free medium, were able to catabolize DON as a carbon source, and produced 3-epi-DON with two undetermined metabolites other than those produced by *Devosia* strains. The level of DON degradation by these strains was similar to that of the *Devosia* strains, though a preliminary incubation in a DON-containing medium was strongly required. The authors suggested these strains possessed some regulatory system for the expression of a DON-degrading enzyme or DON-uptake machineries. Based on the observed difference between the DON-degradation phenotypes of both groups, the authors postulated that the Gram-positive strains represent native DON destroyers, which DON-degrading ability plays a key role for their survival, while Gram-negative bacteria are rather casual DON degraders. Note further sequencing of the *Nocardioides* strain LS1 indicated the lack of genes similar to the earlier described genes encoding DON-degrading enzymes [121] resulting in a conclusion that LS1 possesses a novel DON-degrading pathway.

Another strain, *Nocardioides* sp. WSN05-2, isolated from a wheat field and showed significant growth on the medium containing DON as a sole carbon source, was able to completely remove DON (1000 μg/mL) after 10-day incubation in a DON-containing MSM medium [122]. The study of DON metabolites by HPLC, MS, and NMR showed the presence of two products (3-epi-DON and undetermined compound) in the culture supernatant. Since both products disappeared after prolonged incubation, the authors suggested they are rather intermediates subjected to the further catabolization by the strain. The authors also demonstrated a 90% reduction of the DON content in artificially inoculated wheat grain (2 μg DON per 100 grains) after a 7-day incubation with WSN05-2 preliminarily induced by cultivation on DON-supplemented MSM medium.

Recently Zhang et al. (2021) reported a novel strain, *Nocardioides* sp. ZHH-013, isolated from soil and able to transform DON (168.74 μM) into 3-epi-DON with 80% efficiency within 48 h of incubation at 30 °C [123]. The ultraperformance liquid chromatography-tandem mass spectrometry (UPLC-MS/MS) and HPLC study of DON metabolites showed that DON was catabolized in a two-step mode with a formation of 3-keto-DON followed by its conversion into 3-epi-DON, which further disappeared.

***Paradevosia shaoguanensis***. The Gram-negative *P. shaoguanensis* strain DDB001 isolated from the wheat field soil was reported to be able to completely degrade DON (200 μg/mL) already after an 8 h incubation at 30 °C in the MSM medium [124]. The LC-MS analysis of DON transformation products revealed the formation of 3-epi-DON.

***Desulfitobacterium* sp.** A novel bacterial strain able to de-epoxify DON was recently isolated from a wheat field soil and identified as *Desulfitobacterium* sp. PGC-3-9 [125]. After a 24 h incubation under anaerobic or aerobic conditions at 37 °C in the minimum medium containing yeast extract, pyruvate, and fumarate (MMYPE medium) supplemented with 500 μg/mL DON, the level of a DON degradation reached 99 and 95%, respectively. The formation of dE-DON via the loss of one of the oxygen atoms of the DON molecule was confirmed by GC-MS. Further study of wheat grains naturally contaminated with DON (11.2 μg/g) showed a similar degradation pattern for both aerobic and anaerobic conditions; after 48 h, 92% of the DON was degraded. These results are interesting, since the studied soil bacterium strain demonstrated the target activity under both aerobic and anaerobic conditions in a wide temperature and pH range, and the DON transformation pathway was rather typical for anaerobic ruminal microorganisms. Note that the strain was also able to de-epoxify several other trichothecene mycotoxins (HT2, nivalenol, and 15-ADON).

***Bacillus licheniformis***. *B. licheniformis* strain YB9 isolated from moldy soil was able to destroy more than 82% DON added to the liquid MSM medium at the concentration of 1 mg/L after a 48 h incubation at 37 °C [126]. Though the authors have not identified the DON degradation products at the moment of publication, they showed YB9 could grow using DON as a sole carbon resource, thus suggesting the DON removal from the medium occurred mainly through enzymatic degradation. Intragastrical administration of YB9 resulted in the restoration of some biochemical and histopathological parameters of mice fed with a DON-containing diet to the levels of the control group and the normalization of their intestinal microflora. Interestingly, the strain also showed strong survival and DON-degrading activity in a simulated gastric fluid, i.e., it may be a good probiotic additive.

***Pelagibacterium halotolerans*.** In 2020, a novel bacterial strain, *Pelagibacterium halotolerans* ANSP101, isolated from a seawater sample and capable of transforming DON to 3-keto-DON by the oxidation of the C3 group was reported [127]. The DON degradation capability of this strain was attributed to intracellular proteins or enzymes present in the cell lysate and reached 80% after a 12 h incubation at 28 °C. The degradation was efficient over a broad range of temperatures (30–40 °C) and pH (8.0–10.0) with the optimum at 40 °C and pH 8.0.

***Sphingomonas* sp**. A strain *Sphingomonas* sp. S3-4 isolated from soil was reported to be able to catabolize DON [128]. The strain was able to completely eliminate DON from the cultivation medium and infected wheat grain (100 μg/mL and 112 μg/g, respectively) after 72 h of incubation at 28 °C. Identification of the DON degradation products by GS-MS and NMR indicated the strain S3-4 sequentially catabolized DON into two compounds, 3-oxo-DON (3-keto-DON) and 3-epi-DON.

***Citrobacter freundii***. A very recent study describes a novel DON-transforming bacterium, *Citrobacter freundii*, isolated from rice root-linked soil [129]. After a 72 h incubation at pH 7 and 37 °C, this plant rhizobacterium successfully transformed >93.5% of DON (10 μg/mL) into 3-keto-DON and DOM-1 identified by HPLC and UPLC-MS/MS. The authors also demonstrated that the DON-transforming ability of this bacterium remained quite high (>60%) under acid conditions, mimicking the gastrointestinal tract environment (72 h incubation at pH 5 and 37 °C). Though the enzymes involved in this process are still under study, the fact of formation of DOM-1 by an aerobic bacterium is interesting and may probably result in the discovery of some new enzymes potentially promising for the mycotoxin decontamination.

***Pseudomonas* sp. + *Lysobacter* sp. mixed culture**. An interesting study describing a mixed culture of two soil-derived bacteria capable of efficiently transforming DON into 3-epi-DON was recently published [130]. After a screening of 85 soil samples using media supplemented with 50 μg/mL of DON as the sole carbon source, the authors obtained two strains, *Pseudomonas* sp. Y1 and *Lysobacter* sp. S1, which 60 h co-incubation with DON at 30 °C resulted in a complete disappearance of the toxin via its two-step transformation into 3-epi-DON with the further disappearance of the last one after 84 h of incubation. At the same time, no significant transformation ability was observed for each strain alone. Thus, the authors concluded about a co-metabolism of Y1 and S1 in the DON transformation process. Further study showed the target activity in cell-free supernatants of the mixed culture (100% degradation of DON within 48 h) indicating the involvement of extracellular enzymes.

Information about anaerobic and aerobic bacteria with the confirmed ability to irreversibly transform DON is summarized in Table 3.

#### 3.2.3. DON-Transforming Enzymes

As in the case of ZEN-transforming microorganisms, the majority of the studies focused on DON biotransformation by microorganisms did not achieve identification of the corresponding enzymes. Based on the known products of irreversible DON transformation to less toxic metabolites (see above), the enzymatic degradation of DON by bacteria could occur by two pathways: (a) aerobic DON oxidation into 3-keto-DON via oxidation of the C3 hydroxyl group along with isomerization to 3-epi DON, and (b) anaerobic de-epoxidation into DOM-1. As far as the authors know, only enzymes responsible for the first transformation pathway have been revealed, whereas enzymes responsible for the DON to DOM-1 transformation still remain unknown. Below, we summarize all known enzymes reported by various authors.

After revealing a DON-transforming strain *Sphingomonas* S3-4, a comparative genomic analysis of this strain combined with a functional screening of an S3-4 genomic BAC library was performed, and a novel member of the aldo/keto reductase superfamily, AKR18A1 responsible for the DON oxidation into 3-keto-DON was revealed [128]. Recombinant AKR18A1 expressed in *E. coli* oxidized DON in the presence of an NADH, but not NADPH. The enzyme maintained the target activity in a wide range of pH (7–11) and temperatures (10–50 °C) and possessed the highest activity at pH 10.6 and 45 °C. However, recombinant AKR18A1 seemed to have the preferable catalytic direction towards the reverse reduction reaction (3-keto-DON to DON) under in vitro assay conditions that may be explained by the lack of an enzyme transforming 3-keto-DON into 3-epi-DON and shifting the equilibrium to the desired side in the recombinant strain. Such enzymes responsible for the second DON catabolization stage remained unknown; nevertheless, AKR18A1 can be considered as a possible component of enzymatic preparations providing a two-step DON transformation.

Another bacterium possessing the ability to a two-step transformation of DON into 3-keto-DON and then 3-epi-DON is *Devosia mutans* strain 17-2-E-8 [89,90]. The further study of this process provided a possibility to identify the first-stage enzyme in this pathway, a dehydrogenase responsible for the selective oxidation of DON at the C3 position [131]. This enzyme, called DepA, was isolated and purified. Combining RNA-sequencing and comparative genomics, the authors evidenced the presence of a redox cofactor pyrroloquinoline quinone (PQQ) and Ca^2+^ are important for the DepA activity: after 60 h incubation of purified DepA with 50 μg/mL DON in the presence of both cofactors, the 3-keto-DON content exceeded 30 μg/mL After codone optimization and cloning in *E. coli*, the resulted DepA (5 μg) completely transformed 100 μg/mL DON into 3-keto-DON after an overnight incubation in the presence of 100 μM PQQ. Note DepA remained stable for at least 1 month at 4 °C as well as after a heat treatment at 60 °C which is important for its possible practical application. Later, the same team also identified DepB, the second-step enzyme reducing 3-keto-DON into 3-epi-DON [132]. The enzyme was classified as NADPH-dependent dehydrogenase and was also characterized by moderate thermostability maintaining its activity after a heat treatment at 55 °C. DepB worked well in a wide pH range (5–9) and in a number of buffers. However, recombinant enzymes from these two sequential reactions have not been shown to work together to complete the DON epimerization pathway.

A genome analysis of another highly active *Devosia* sp. strain D6-9 resulted in the identification of three genes involved in the DON epimerization process [119]. One gene encoded a PQQ-dependent DON dehydrogenase (QDDH), which oxidized DON into 3-keto-DON in the presence of PQQ and Ca^2+^ in wide pH (5–8) and temperature (20–55 °C) ranges with the highest activity at pH 6 and 40 °C. This enzyme was shown to have 100% similarity to DepA. Two other genes encoded NADPH-dependent aldo/keto reductases (AKR13B2 and AKR6D1) converting 3-keto-DON into 3-epi-DON. These two enzymes were also characterized by wide working pH and temperature ranges. The mix of the corresponding recombinant enzymes expressed in *E. coli* completely converted 100 μM DON into 3-keto-DON and 3-epi-DON after 4 h of co-incubation in a buffer. Application of these enzymes on flour prepared from wheat grain infected with pathogens causing Fusarium head blight showed a 99.3% DON reduction after 6 h of incubation. The authors of the study also revealed the role of S497, E499, and E535 residues in the DON-oxidizing activity of QDDH.

Another DON dehydrogenase (DDH) possessing DON oxidation activity was identified in *Pelagibacterium halotolerans* ANSP101 by a comparative genome analysis [133]. This enzyme was not dependent on PQQ, but required other hydrogen acceptors, phenazine methosulfate and dichlorophenolindophenol. The authors of the study improved DDH by a site-specific mutagenesis, cloned and expressed the wild and mutant (TDDH) enzymes in *E. coli*, and evaluated their DON-transforming capacity. The degradation rates of enzymes reached 85–87%, but TDDH exhibited stronger degradation ability for DON compared with wild DDH; in the presence of PQQ, TDDH efficiently degrades DON into 3-keto-DON in a wide pH (6.0–11.0) and temperature (20–45 °C) range. The authors also mentioned TDDH showed better kinetic parameters than AKR18A1, DepA, and QDDH.

## 4. Biotransformation of Fumonisin B1

### 4.1. Fumonisin B1 Biotransformation Pathway

**Figure 5:**
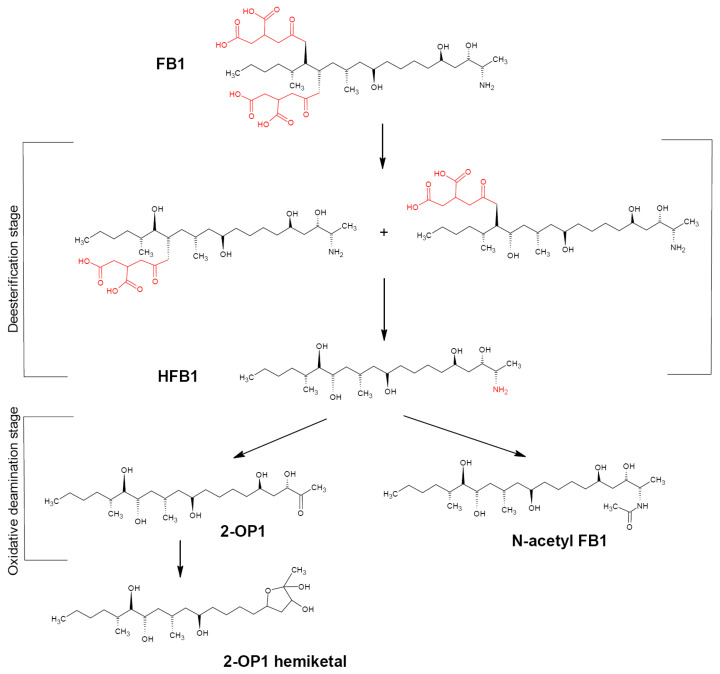
Two-step fumonisin biotransformation pathway. Functional groups modified or cleaved at each stage are indicated in red.

FB1 represents a chemically stable diester compound. The FB1 skeleton is a fat chain consisting of 20 carbon atoms and two tricarballylate (TCA) side groups connected to C14 and C15 atoms. These two chains as well as a free amino group are important for the FB1 toxicity since their removal significantly reduces the toxicity effects; thus, at least two enzymatic activities are necessary for efficient FB1 detoxification [134]. The number of microorganisms able to degrade FB1 to reduce its toxicity is rather low. Such microorganisms are capable of using FB1 as the sole carbon source and transforming it into two steps (Figure 5) [13]. First, TCA groups at C14 and C15 are cleaved by fumonisin esterase to form hydrolyzed FB1 (HFB1) characterized by a significantly lower toxicity compared to FB1. The process is two-staged with a formation of two intermediates. Second, HFB1 is degraded by aminotransferase either to 2-keto-HFB1 (2-OP1) with the further irreversible cyclization to 2-OP1 hemiketal [134,135] or N-acetyl HFB1 that can potentially revert to HFB1 through amide hydrolysis [99]. Alternatively, a direct deamination of native FB1 with fungal amine oxidases with a formation of deaminated FPy and FLa fumonisins is possible [136,137,138].

### 4.2. FB1-Transforming Microorganisms

To date, the vast majority of studies reporting FB1-degrading activity of microorganisms include no data on the in vitro confirmed transformation efficiency (i.e., data confirming the reduced toxicity of biotransformation products or their identity with the compounds with the proved and irreversible loss of toxicity). Taking into account the aim of this review, this subsection describes only studies, in which the authors confirmed the presence of the enzymes proved to be involved in the FB1 detoxifying activity in the studied microorganisms. The list of such microorganisms is presented in Table 4.

#### 4.2.1. FB1-Transforming Bacteria

***Sphingomonas* and *Sphingopyxis* spp.** Microorganisms able to degrade fumonisins include *Sphingomonas* widely occurred in aquatic and terrestrial environments. *Sphingomonas* sp. ATCC 5552 isolated from moldy maize grain was shown to degrade FB1 via consecutive action of two enzymes, carboxylesterase and aminotransferase [139,140]. These degradation pathways were confirmed by TLC and radiochemical methods. The same pathway was also shown for the MTA144 strain belonging to the related genus *Sphingopyxis* and isolated from moldy soil [133]. The authors of the above-mentioned studies (Refs. [134,140]) isolated genes responsible for these enzymes and obtained the corresponding recombinant enzymes, which target activity was confirmed by LS-MS analysis (see Section 4.2.3).

***Delftia/Comamonas* sp.** A screening of soil samples for FB1-degrading microorganisms performed using an enrichment culture procedure resulted in the isolation of the NCB 1492 strain belonging to Gram-negative bacteria tentatively assigned to the *Delftia/Comamonas* group [141]. According to TLC results, the strain was able to completely degrade 0.5 mg/mL of FB1 used as the sole carbon source after 24 h of incubation. The HPLC analysis of the degradation dynamics showed the disappearance of the FB1-associated peak already after 30 min of incubation, with the appearance of another peak also disappearing after 2 h. A GS-MS analysis of degradation products in a culture supernatant performed after 24 h of incubation revealed the presence of four compounds, identified as heptadecanone, isononadecene, octadecenal, and eicosane. Some additional experiments allowed the authors to suggest the NCB 1492 possessed deamination and (possibly) esterase activity.

***Serratia marcescens***. Screening of 95 samples from natural sources for FB1-degrading activity resulted in the identification of a *Serratia marcescens* strain 329-2 possessing the target activity [142]. The FB1 reduction by the cell-free extract of the strain 329-2 reached 30.29% under in vitro conditions and 37% in the case of treatment of contaminated maize grain (in both cases, FB1 concentration was 5 μg/mL, and incubation time was 24 h). The residual FB1 concentration was determined by ELISA assay. The MS-based proteomic analysis revealed upregulated expression of a number of proteins, among which alpha/beta hydrolase (A0A656VL53) and acetylornithine/succinyldiaminopimelate aminotransferase (A0A6H1E4N5) represented potential enzymes associated with FB1 degradation according to the same scenario as described for other FB1-degrading bacteria.

**Bacterial consortium SAAS79**. A group of bacteria (consortium SAAS79) efficiently degrading FB1 was isolated from the spent mushroom compost [143]. The consortium consisted mainly of *Pseudomonas* species (86.4%), which was shown to play a major role in the FB1 degradation. In addition, SAAS79 included *Delftia* and *Sphingobacterium* genera previously reported as FB1 transformers. Unfortunately, the authors did not succeed in the isolation of any active strain from the consortium, so they supposed a synergistic effect between the species composing SAAS79. According to the results of the LS-MS/MS analysis, the degradation rate of 50 µg/mL of FB1 reached 100% after 24 h incubation at 28 °C. Further experiments proved the key role of intracellular crude enzymes in FB1 degradation; 20 µg/mL of FB1 was destroyed by ~90% after 3 h incubation at 28 °C. Interestingly, the enzymes showed a high specificity towards fumonisins: incubation with 10 µg/mL of AFB1, DON, ZEN, T2, FB1, and FB2 resulted in >80% destruction of FB1 and FB2 only. Identification of degradation products using the liquid chromatography time-of-flight mass spectrometry (LS-TOF/MS) indicated the presence of two metabolites, one of which corresponded to the loss of a TCA group by FB1 (pHFB1a or pHFB1b), and the second corresponded to HFB1, i.e., to the loss of both TCA groups. A cytotoxicity study using an MTT assay and MARC-145 cell culture showed no significant difference in cell viability between the control and 10 μM FB1 degradation products, but a significant drop in cell viability for the FB1-treated variant. Due to a high degradation efficiency and a wide working temperature range (20–50 °C), SAAS79 probably has a high potential for application in the feed and food industry.

#### 4.2.2. FB1-Transforming Fungi and Yeasts

***Exophiala spinifera***. One of the first fungi known for their FB1-degrading ability is *Exophiala spinifera* strain 2141.10, a black yeast fungus isolated from moldy maize ears and capable of using FB1 as a sole carbon source [135]. The degradation process includes enzymatic FB1 hydrolysis of TCA side chains by fumonisin tricarballylate esterase producing free tricarballic acid and an aminopentol backbone. The further metabolization of hydrolyzed FB1 involves oxidative deamination by the induced *E. spinifera* culture with a formation of two derivatives, N-acetyl HFB1 (minor compound) and 2-OP1 hemiketal determined by NMR and MS studies. Both compounds lose a free amino group crucial for the toxicity of fumonisins.

***Aspergillus* sp**. During a study of mycotoxin-producing *Aspergillus* species in Canadian wineyards, two new types of deaminated fumonisins, FPy_4_ and FLa_4_, were identified from cultures of *A. welwitschiae* [136]. Further elucidation of the structure of these fumonisins as well as the evaluation of their toxicity using a *Lemna minor* bioassay showed these compounds were less toxic than FUM mycotoxins [137]. The study of their production by *A. welwitschiae* strain DAOMC 250,207 using LS-MS, NMR, and stable isotope labeling resulted in a suggestion of a novel post-biosynthetic oxidative deamination process for fumonisins. The examination of this hypothesis by a feeding study with FB1 as a sole carbon source demonstrated biotransformation of FB1 to FPy_1_ and confirmed some *Aspergillus* species have the ability to produce enzymes naturally capable of detoxifying fumonisins by oxidative deamination. Later, the same research team confirmed the presence of similar enzymes in *A. niger* fungi (see Section 4.2.3) [138].

#### 4.2.3. FB1-Transforming Enzymes

To date, several enzymes involved in the FB1 degradation have been identified. In both bacteria and fungi/yeasts, the process starts with the hydrolytic release of the two tricarballylic acid side chains catalyzed by a fumonisin carboxylesterase. The second step (HFB1 deamination) involved either amine oxidase (fungi/yeasts), or aminotransferase (bacteria).

**Fumonisin carboxylesterases**. To date, the majority of fumonisin carboxylesterases were revealed in bacteria belonging to the family *Sphingomonadaceae*. A *fumD* gene encoding carboxylesterase was isolated from *Sphingopyxis* sp. MTA144 and heterologously expressed in *Pichia pastoris* [134]. The recombinant carboxylesterase (18 U/L) was shown to completely convert 60 μg/mL of FB1 to HFB1 after a 15 min co-incubation at 30 °C and pH 8.0 [144] completely degraded 4.63 μM FB1 to HFB1 within 15 min at 30 °C. The enzyme was commercialized by the BIOMIN company as FUMzyme^®^ and successfully authorized by EU for use as a feed additive enabling gastrointestinal deesterification of FB1 in pigs and poultry [145]. Further studies of this preparation confirmed its high efficiency as a feed additive in pigs, turkeys, and chickens [146] as well as a significant (>80%) reduction of the FB content in the maize grain after a 1 h treatment [147].

The study [139] revealed the *Sphingomonas* sp. strain ATCC 5552 possessed FB1-transforming activity. Further studies resulted in the identification of a corresponding carboxylesterase (BacEst); the authors of the study claimed and patented the nucleotide sequence corresponding to the detoxification esterase of the above-mentioned strain and expression cassettes for the transformation of various host cells and organisms [148]. However, we did not find other mentions about this enzyme.

A novel fumonisin-degrading carboxylesterase was revealed by the gene mining analysis using a FumD from *Spingopyxis* sp. MTA144 as a reference to find similar proteins [149]. The resulting recombinant enzyme (FumDSB) from *Sphingomonadales bacterium* KUO56785 was cloned and expressed in *E. coli*. Examination of its detoxification activity by coupled tandem mass spectrometry showed the enzyme efficiently cleaved two TCA chains of FB1; the specific activity of FumDSB towards FB1 was 1.12 U/mg at 30 °C and pH 6.0 corresponding to the physiological pH of animal intestine (5.0–6.0). This activity was maintained within a broad pH range (from 6.0 to 9.0). Further studies proved the efficiency of FumDSB in alleviating FB1-induced inflammatory responses in pigs [150].

Along with the above-mentioned enzymes from *Sphingomonadaceae* bacteria, there is also information about fumonisin esterase AP1 from the black yeast *Exophiala spinifera* (see [135]) also cleaving TCA chains from FB1. The authors of the mentioned study succeeded in the further identification of the enzyme-encoding nucleotide sequence followed by its patenting [139].

**Aminotransferases**. An aminotransferase-encoding *fumI* gene was isolated from *Sphingopyxis* sp. MTA144 and heterologously expressed in *E. coli* [134]. The recombinant enzyme showed a significant HFB1 deaminating activity degrading about 90% of initial FHB1 (5 mg/L equal to 12.4 μM) within 44 h incubation at 25 °C. Interestingly, the process did not depend on the presence of molecular oxygen, but required pyruvate or other α-keto acids as a co-substrate for aminotransferase activity. This property provides the possibility of applying FumI to the treatment of ensilaged forage or as a feed additive supplemented with pyruvate for gastrointestinal FB1 detoxification. The enzyme showed activity in the range of pH 6 to 10 with an optimum at pH 8.5, and in the range of 6–50 °C with an optimum at 35 °C [151]. Based on the enzyme characteristics, the authors concluded that the technological application of FumI, in combination with the fumonisin carboxylesterase FumD for hydrolysis of fumonisins, for deamination and detoxification of hydrolyzed fumonisins seems possible if the enzyme properties are considered.

The same authors also studied the recombinant aminotransferase from the bacterial ATCC 55,552 strain in *E. coli* and confirmed its target activity [140]. A crude lysate of the recombinant strain in a phosphate buffer showed nearly complete deamination of 8.9 μM HFB1 after 15 h incubation at 25 °C in the presence of the α-keto acid pyruvate or coenzyme pyridoxalphosphate. As in the case of MTA144, this enzyme did not depend on the presence of molecular oxygen that could be advantageous for some technological applications in the future.

The authors of a very recent study performed gene mining to construct three novel fumonisin transaminases (aminotransferases) [152]. These enzymes called FumTSTA, FumUPTA, and FumPHTA were from *Paraburkholderia madseniana* (FumTSTA) and *Phenylobacterium* sp. (FumUPTA, and FumPHTA) and shared only 61–74% sequence identity with other reported fumonisin aminotransferases. They were cloned and expressed in *E. coli*. The FB1-degrading assay included incubation of the purified recombinant enzymes with HFB1 (1 μg/mL) obtained by the FB1 treatment with FumDSB esterase for 12 h at 37 °C. All three enzymes were able to completely degrade HFB1 into 2-OP1, but displayed different optimal pH (9.0, 8.0, and 4.0) that significantly enlarged their application scenes for either individual or combined use. Moreover, all enzymes showed good thermostability. The broad pH and temperature range may expand the application aspects of the reported enzymes.

**Amine oxidases.** Garnham et al. [138] identified and characterized an enzyme from *Aspergillus niger* providing an alternative way for direct FB1 deamination with a formation of deaminated fumonisin FPy_2_. The enzyme called AnFAO belonged to the monoamine oxidase family. It was cloned and expressed in *E. coli* with the further demonstration of its ability to oxidatively deaminate intact fumonisins without any exogenous cofactors. The authors showed that 1 h incubation of 6 nM homogeneous AnFAO with 1 μM FB1 at 37 °C resulted in a complete conversion of the intact substrate into FPy_2_. Among the advantages of this enzyme, the authors mentioned a broad range of working conditions, a high native melting temperature, and a high-yield production. Moreover, unlike bacterial aminotransferases, AnFAO does not require expensive cofactors, such as pyridoxal phosphate or pyruvate and also does not require upstream carboxylesterases thus representing a promising tool to remediate fumonisin-contaminated feed including maize destined for ethanol production. This study was the first report of a fumonisin detoxifying enzyme produced by the same fungus that synthesizes the toxin; the authors supposed AnFAO may provide protection of a fungus from the toxic effects of its own mycotoxin.

**Fused enzymes**. An interesting study describing a fusion enzyme of carboxylesterase and aminotransferase for FB1 degradation was published in 2021 [153]. The authors of this study designed, constructed, and expressed a recombinant fusion enzyme called FUMDI by linking the carboxylesterase gene (*fumD*) and the aminotransferase gene (*fumI*) by overlapping polymerase chain reaction (PCR). The resulting enzyme was successfully expressed in *P. pastoris* and demonstrated a high biodegradation activity towards FB1 as well as some other FBs, such as FB2 and FB3. FUMDI and almost completely degraded 5 μg/mL of each toxin in a reaction buffer within 24 h at 25 °C sequentially converting it to HB1 and 2-OP1. FUMDI enzyme and its reaction products were shown to have no negative effects on cell viability and did not induce cell apoptosis, oxidative stress, or endoplasmic reticulum (ER) stress in a GES-1 cell line. Thus, the authors concluded FUMDI can be used for the simultaneous and safe control of the FB1, FB2, and FB3 contamination of food and feed products.

## 5. Prospects for Use of Microbial Destructors of Fusariotoxins and Promising Directions of Future Studies

Compared to other mycotoxin degradation methods, the use of microbial cultures for mycotoxins transformation has some advantages, such as the possibility of their large-scale industrial production, the physiological range of required reaction conditions, and their ability to perform multi-step transformation processes. At the same time, the use of live cell cultures has some drawbacks, such as the production of undesirable metabolites, changes of organoleptic characteristics, or even spoilage of the treated products, as well as possible changes in the culture growth, physiology, and efficiency of mycotoxin biotransformation depending on the working conditions, level of mycotoxin contamination, and the presence of other microbes in the environment. Note also that, in many cases the revealed microorganisms may transform mycotoxins via reversible pathways, resulting in a threat of the recovery of such modified toxins and the associated toxicity of treated food products. From this point of view, a full understanding of the structure and stability of degradation products is very important for evaluating their potential side-effects and safety.

Mycotoxin transformation by enzyme preparations makes it possible to avoid these drawbacks. Enzymes provide more reproducible and easily handled performance, lack contamination risks, and have no safety concerns; their reaction pathways are usually well-studied. They can be manufactured by large-scale fermentation, including the use of genetic engineering for cloning and expressing heterologous enzymes in microbial producers or plant cells for less expensive and less laborious production. Last but not least, the identification and characterization of mycotoxin-degrading enzymes provide elucidation of transformation pathways and the possibility of choosing those of them, which provide irreversible degradation with a formation of less or non-toxic metabolites.

To date, in spite of numerous studies in the described area, the number of commercialized fusariotoxin-degrading microbial products is very low and includes only several preparations, all registered by the BIOMIN company. One of them, Biomin^®^ BBSH 797, represents the strain of anaerobic *Eubacterium* sp. BBSH 797 able to transform DON to DOM-1 in the gastrointestinal tract of cattle [110]. The second is Biomin^®^ MTV, a non-pathogenic yeast *Trichosporon mycotoxinivorans* able to degrade ZEN and ochratoxin A. Two others are enzyme preparations and include ZENzyme^®^, a ZEN hydrolase derived from *Rhodococcus erythropolis* PFA D8-1 [79,80] and FUMzyme^®^, a recombinant FUM carboxylesterase from *Sphingopyxis* sp. MTA144 [145]. In addition, the company developed six variants of a combined Mycofix^®^ preparation (initially consisting of lyophilized biomass of two microbial strains, *Bacterium* BBSH797 for degradation of DON and related trichothecenes and *T. mycotoxinivorans* for degradation of ZEN and ochratoxin A) to protect cattle and poultry against various ranges of mycotoxins. Note the results of the use of some commercial feed additives can be controversial in relation to their efficiency. For example, Dänicke et al. [154] reported the Mycofix^®^Plus preparation was ineffective in preventing chronic DON toxicosis in pigs since no positive effects on performance and no alterations in DON concentration in serum or excretion of DON with the urine were observed. Thus, the studies in this field intended to fill the niche of efficient mycotoxin-degrading preparations still remain relevant.

### 5.1. Promising Directions of Studies Intended to Search for Potential Microbial and Enzymatic Agents Able to Transform Fusariotoxins

Taking into account the aforesaid, the main emphasis in the search for microbes able to degrade mycotoxins should be made on the selection of strains capable of irreversibly transforming such compounds via the known pathways with further identification of the corresponding enzymes and obligatory toxicity tests, since such an approach will make it possible to focus on strains (and then enzymes) with good commercialization potential.

Based on the existing publications, a good way to search for new sources of mycotoxin-degrading enzymes may be a screening of microbial strains for the ability to utilize mycotoxins as the sole carbon source, since in this case the possibility of their irreversible conversion into less toxic metabolites is rather high [54,55,122,126,130]. Another possible way is a screening of microbial strains belonging to the genera that provided some effective mycotoxin-transforming strains. For example, a number of ZEN-degrading strains were revealed among the genus *Rhodococcus* [50,51,52,53], and a number of DON-degrading strains were reported among the genera *Devosia* [115,117,118,119,120] and *Nocardioides* [120,121,122,123]. In the case of ZEN, good screening results may be also obtained using some biotests providing an evaluation of the estrogenic potential of ZEN transformation products [50]. Finally, the search for possible new mycotoxin-degrading microorganisms and enzymes can be now performed using bioinformatic tools and available genomic and protein databases. Such an approach is described in detail in the study [155] and may include a search for genes similar to those encoding the known mycotoxin-degrading enzymes, the use of structure-to-function reductive filter models to work with enzymes sharing functional, but not sequence similarity, and molecular docking to determine binding pockets and evaluate the affinity level of a possible enzyme-substrate interaction. The revealed candidate enzymes should be then tested for their real substrate-degrading activity and degradation products. As examples, we can mention the study [149], where the authors used gene mining to reveal a novel carboxylesterase FumDSB from *Sphingomonadales bacterium*, and the study [152] resulted in the revealing of three novel fumonisin aminotransferases FumTSTA, FumUPTA, and FumPHTA from *Paraburkholderia madseniana* and *Phenylobacterium* sp. In some cases, additional computational tools can be used. For example, in the case of secreted enzymes requiring a signal peptide to move across the cell membrane, the use of special algorithms to identify such sequences and the secretion pathway may be useful for the identification of candidate mycotoxin-degrading enzymes. For example, the use of a SignalP algorithm facilitated the search of gene candidates for the DON-degrading DepA enzyme in *Devosia mutans* [131].

Computational tools can be also used for codon optimization that improve expression of recombinant enzymes [131] and for the rational design of native enzymes to improve their specificity towards target substrates, expand the substrate range, or optimize their degradation potential [156]. For example, the Val153His replacement in a ZHD enzyme did not change its activity towards ZEN, but provided a 3.7-fold increase in the activity of an engineered enzyme towards α-ZEL [157].

### 5.2. Future Directions of Studies to Solve Issues Related to the Practical Application of Fusariotoxin-Transforming Bioagents

A large number of issues should be answered in relation to the practical aspects of the application of fusariotoxin-transforming microorganisms and enzymes. Microorganisms, if not considered as the source of mycotoxin-degrading enzymes, are usually considered for use as feed additives to degrade mycotoxins in the gastrointestinal tract of cattle and poultry. In this case, they should belong to the known probiotic microbes and maintain the target activity at low pH, which narrows the range of suitable strains. As an example of such a strain, one can mention *B. licheniformis* YB9 [126] that showed a high survival degree and activity at low pH values. In the case of enzymes, the resulting preparations can be aimed at the use as either feed additives, or preparations for the treatment of stored feeds. In both cases, there is a need to create stable forms of these enzymes to prevent their deactivation under an aggressive environment.

Enzyme stability and tolerance to the solvents and environments can be improved by in silico approaches combined with targeted mutagenesis. For example, the modification of two amino acid residues at positions 134 and 136 of the ZENG lactonase from *Clonostachys rosea* significantly improved its thermostability compared to the wild-type enzyme [70]. Another way to increase the stability of enzymes is their encapsulation with controlled release or immobilization. For example, the immobilization of the recombinant dissolved ZHD101 lactonase (rdZHD) from *Clonostachys rosea* IFO 7063 using a cross-linked poly(γ-glutamic acid)/gelatin hydrogel had better thermostability and a broader pH range with higher enzyme activity than free rdZHD [158]. Immobilization can also improve the target efficiency of enzymes. Guo et al. [86] reported that immobilization of a CotA laccase from *Bacillus licheniformis* on chitosan particles increased its ZEN degradation efficiency from 70 (free CotA) to 90%.

Since agricultural products are often contaminated with several mycotoxins, the search for compounds able to provide degradation of several mycotoxins represents a significant research task. To date, a number of native strains and enzymes are known to degrade several different mycotoxins. For example, the *Eubacterium* sp. strain (Biomin^®^ BBSH 797) was shown to degrade several trichothecene mycotoxins structurally related to DON [110]. *Desulfitobacterium* sp. PGC-3-9 was able to de-epoxify DON and some other trichothecene mycotoxins, such as nivalenol, HT-2, and 15-ADON [125]. Extracellular metabolites from *Clonostachys rosea* GRZ7 demonstrated both AFB1- and ZEN-degrading activity [58,59]. The same ability was reported for a number of laccases and peroxidases (see [84,159]). Further researches may reveal some novel enzymes possessing such multi-degrading ability. At the same time, a new effective approach has recently appeared, which can be used to create multi-functional fused enzymes with an extended substrate range. A FUMDI enzyme combined the properties of the carboxylesterase FumD and aminotransferase FumI and provided irreversible degradation of FB1, FB2, and FB3 [153]. A ZPF1 enzyme with the ability to degrade AFB1 and ZEN was constructed via the linking of mutant V153H of ZHD101 and manganese peroxidase [160]. A fused enzyme ZHDCP was obtained by combining zearalenone hydrolase (ZHD) and carboxypeptidase (CP) and was able to degrade ZEN and ochratoxin A (OTA) [161]. In spite of interesting results reported by the authors, there are still some concerns about the degradation efficiency of such fusion enzymes compared with that of single enzymes [160].

## 6. Conclusions

The biological degradation of fusariotoxins using microbial strains or their enzymes is a promising environmentally-friendly alternative to the chemical and physical detoxification methods. To date, there are a number of reports describing microorganisms with the target activity against mycotoxins; however, not all of them include the study of the mechanisms of such biotransformations, identification of degradation products, evaluations of the transformation efficiency not only in model solution but also under in vivo conditions as well as in vivo safety evaluation. At the same time, this information is very important for the search for potential sources of efficient fusariotoxin-degrading enzymes suitable for further commercialization and practical application; thus, the authors hope this review will be useful for our colleagues working in this field of science.

## Figures and Tables

**Table 1:** Occurrence of zearalenone, deoxynivalenol, and fumonisins in feed and raw commodity samples from different regions of the world (January–September 2021) *.

Region	Fusariotoxin Occurrence, % of Samples		
	Zearalenone	Deoxynivalenol	Fumonisins
Europe	46	52	40
Middle East	65	58	90
Pacific Asia **	71	80	90
North America **	32	69	73
Central America	73	68	-
South America	40	46	66
Africa	44	81	51

* Data were calculated based on 76,300 analyses of 16,164 finished feed and raw commodity samples originating from 74 countries [4]. ** The data are shown for corn samples only.

**Table 2:** Microbial species able to efficiently transform zearalenone (ZEN) into nontoxic compounds.

Species	Type of Experiment and ZEN Concentration	ZEN Degradation Level, %	Approaches Used for Evaluation of Biotransformation Efficiency/Mechanism of Transformation	Data From
***Bacteria***				
*Bacillus subtilis 168*	In Vitro, LB medium; 20 μg/L	81	MS analysis (lack of estrogenic-type metabolites)/**Unknown (includes decarboxylation)**	[44]
*B. natto* CICC 24640	In Vitro, LB medium; 20 μg/L	100	MS analysis (lack of estrogenic-type metabolites)/**Unknown (includes decarboxylation)**	[44]
*B. pumilis* ES-21	In Vitro, LB medium; 17.9 μg/mL	95.7	LC-TOF-MS/MS analysis (non-estrogenic metabolite)/**Lactone ring cleavage**	[45]
*B. velezensis* ANSB01E	In Vitro, LB medium; 2 μg/mL	95	BLAST analysis (revealing of genes encoding proteins similar to confirmed ZEN-transforming enzymes)/**Unknown (possibly cracking of a dihydroxybenzene ring)**	[46]
	In Vitro, corn meal medium; 0.74 μg/mL	~75		[46]
*Rhodococcus ruber* N361	In Vitro, LB medium; 2 μg/mL	60	ELISA test for ZEN metabolites, BLYES bioassay for estrogenicity (40–60% reduction)/**Unknown**	[49]
*R. erythropolis* NI1	In Vitro, LB medium; 2 μg/mL	50	ELISA test for ZEN metabolites, BLYES bioassay for estrogenicity (40–60% reduction)/**Unknown**	[49]
*R. pyridinivorans* K402	In Vitro, LB medium; 2 μg/mL	70.11	ELISA test for ZEN metabolites, BLYES bioassay for estrogenicity (100% ceasing)/**Unknown**	[49]
*R. pyridinivorans* K404	In Vitro, LB medium; 2 μg/mL	72.3	ELISA test for ZEN metabolites, BLYES bioassay for estrogenicity (100% ceasing)/**Unknown**	[49]
*R. pyridinivorans* K408	In Vitro, LB medium; 2 μg/mL	80.3	ELISA test for ZEN metabolites, BLYES bioassay for estrogenicity (100% ceasing)/**Unknown**	[49]
	In Vitro, LB medium; 5 μg/mL	87.2	BLYES bioassay (81.75% reduction of estrogenicity)	[50]
	In Vivo, female rats	-	Immature uterotrophic assay (lack of estrogenicity)	[51]
*R. percolatus* JCM 10087T	In Vitro, LB medium; 1 μg/mL	95	BLYES bioassay for estrogenicity (70% reduction)/Unknown	[52]
*R. erythropolis* PFA D8-1	In Vitro	-	Bioassays with the MCF cell line and estrogen reporter yeast strain YZHB817 (estrogenicity), metabolite identification/**Lactone ring cleavage**	[53]
*Acinetobacter* sp. SM04	In Vitro, M1 medium; 20 μg/mL; In Vitro, MCF-7 cells	~100	HPLC and MS for ZEN metabolites; MTT cell proliferation assay for estrogenic activity (no estrogenic effect)/**Cracking of a dihydroxybenzene ring**	[54]
*Pseudomonas* sp. ZEA-1	In Vitro, M1 medium; 100 μg/mL	100	TLC analysis for ZEN metabolites/**Unknown**	[55]
	In Vivo, *Artemisia salina* larvae	-	Toxicity tests	[55]
***Fungi* and *yeasts***				
*Clonostachys rosea (Gliocladeum roseum)* NRRL1829	In Vitro, 250 μg/mL	80–90	MS, NMR, and IR spectral analysis of ZEN metabolites; inhibition of estradiol binding to estrogen receptors to evaluate estrogenic activity/**Lactone ring cleavage**	[56]
*C. rosea* IFO 7063	In Vitro, 100 μg/mL	nd *	NMR and MS analysis of ZEN metabolites; MCF-7 cell culture assay to evaluate estrogenic activity/**Lactone ring cleavage**	[57]
*C. rosea* GRZ7	In Vitro, 0.5 μg/mL	68	Isolation of lactonohydrolase with the confirmed target activity/**Lactone ring cleavage**	[59]
*Trichosporon mycotoxinivorans*	In Vitro, saline medium, 10 μg/mL; In Vivo, yeast bioassayIn Vitro, estrogen receptor binding assay	95	LC-MS/MS, LC-DAD, TOF MS, NMR analysis of ZEN metabolites; indicator yeast bioassay and human estrogen receptor protein binding assay for estrogenicity evaluation/**Cracking of a C6′** **ketone carbonyl group**	[65]

* Not described. Here and in the subsequent tables: MS, mass spectrometry; LC-TOF-MS/MS, liquid Chromatography time-of-flight tandem mass spectrometry; BLAST, basic local alignment search tool; ELISA, enzyme-linked immunosorbent assay; BLYES, bioluminescent yeast estrogen; HPLC, high-performance liquid chromatography; TLC, thin-layer chromatography; NMR, nuclear magnetic resonance spectroscopy; IR, infrared spectroscopy; LC-MS/MS, liquid chromatography-tandem mass spectrometry; LC-DAD, LC-diode array detector analysis; TOF MS, time-of-flight mass spectrometry.

**Table 3:** Microbial species able to efficiently transform deoxinyvalenol (DON) into nontoxic compounds.

Species	Experimental Conditions and DON Concentration	DON Degradation Level, %	Approaches Used for Evaluation of Biotransformation Efficiency (Metabolite)	Data From
***Anaerobic bacteria***				
*Bacillus* sp. LS100	In Vitro, AIM medium, 100 μg/mL	100	LS-MS analysis of DON metabolites (**DOM-1**)	[105]
	In Vivo (pigs), 124 μg/g of corn	-	LS-MS analysis of DON metabolites; feed intake, weight gain, and feed efficiency to evaluate toxicity	[106]
*Eggerthella* (*Raoultibacter*) sp. DII-9	In Vitro, BHI broth, 100 μg/mL	100	LC-MS analysis of DON metabolites (**DOM-1**)	[107]
*Slackia* sp. D-G6	In Vitro, BHI broth, 100 μg/mL	>90	HPLC analysis of DON metabolites (**DOM-1**)	[108]
*Coriobacteriaceum* DSM 11798	In Vitro, M10 medium, 100 ppm	100	RP-HPLC and TLC analysis of DON metabolites; LPA assay for toxicity evaluation (**DOM-1**)	[109,111]
*Clostridium* sp. WJ06	In Vitro, L10 medium, 20 ppm	>90	LC-MS/MS analysis of DON metabolites (**DOM-1**)	[113]
	In Vivo (pigs), 1.9 mg/kg of feed	-	Toxicity evaluation (growth performance, intestinal morphology, relative organ weight, intestinal flora)	[113]
***Aerobic bacteria***				
E3-39 isolate (*Agrobacterium–Rhizobium* group)	In Vitro, BYE medium, 200 μg/mL	100	TLC and NMR analysis of DON metabolites; mouse spleen lymphocyte proliferation assay to evaluate immunosuppressive activity (3-keto-DON)	[114]
*Devosia mutans* 17-2-E-8	In Vitro, CMB medium, 100 μg/mL	95	HPLC analysis of DON metabolites (**3-epi-DON**)	[115]
*Devosia insulae* A16	In Vitro, MSM medium, 20 μg/mL	88	HPLC, LC-TOF-MS and NMR analyses of DON metabolites (3-keto-DON)	[117]
*Devosia* A6-243	In Vitro, MSM medium + PQQ, 100 μg/mL	100	QTOF LC–MS and NMR analyses of DON metabolites (**3-epi-DON**)	[118]
	In Vitro, wheat grain, 6.7 µg/g	100	QTOF LC–MS and NMR analyses of DON metabolites (**3-epi-DON**)	[118]
*Devosia* sp. D6-9	In Vitro, 500 μg/mL	100	HPLS and GC-MS identification of DON metabolites (**3-keto-DON** and **3-epi-DON**)	[119]
	In Vitro, wheat grain, 11.5 µg/g	100	HPLS and GC-MS identification of DON metabolites (**3-keto-DON** and **3-epi-DON**)	[119]
*Devosia* sp. SS5, RS1, NKK1, NKJ1	In Vitro, MSM medium, 100 μg/mL	~99.5%	HPLC analysis of DON metabolites (**3-epi-DON**)	[120]
*Nocardioides* sp. PFS1, YMN1, SS1, SS2, SS3, SS4, LS1, LS2, YUL1	In Vitro, MSM medium; 100 μg/mL (pre-incubation with DON)	~99.5%	HPLC analysis of DON metabolites (**3-epi-DON**)	[120]
*Nocardioides* sp. WSN05-2	In Vitro, MSM medium, 1000 μg/mL	100	LC-MS and NMR analyses of DON metabolites (**3-epi-DON**)	[122]
	In Vitro, wheat grain, 2 μg/100 grains	100	LC-MS and NMR analyses of DON metabolites (**3-epi-DON**)	[122]
*Nocardioides* sp. ZHH-013	In Vitro, MSM medium, 168.74 μM	80	LC-MS analysis of DON metabolites (**3-epi-DON**)	[123]
*Paradevosia shaoguanensis* DDB001	In Vitro, MSM medium, 200 μg/mL	100	LC-MS analysis of DON metabolites (**3-epi-DON**)	[124]
*Desulfitobacterium* sp. PGC-3-9	In Vitro, MMYPF medium, 500 μg/mL	99/95 (anaerobic/aerobic)	GC-MS analysis of DON metabolites (**DOM-1**)	[125]
	In Vitro, wheat grain, 11.2 μg/g	92	GC-MS analysis of DON metabolites (**DOM-1**)	[125]
*Bacillus licheniformis* YB9	In Vitro, MSM medium, 1 μg/L	82.67	ELISA (DON Plus Test Kit) for DON quantification, use of DON as a sole carbon source	[126]
*Pelagibacterium halotolerans* ANSP101	In Vitro, MMB2216 medium, 50 µg/mL	80	UPLC-MS/MS analysis of DON degradation products (3-keto-DON)	[127]
*Sphingomonas* sp. S3-4	In Vitro, MSM medium, 100 µg/mL	100	GC-MS and NMR analysis of degradation products (**3-oxo-DON (=3-keto-DON)** and **3-epi-DON**)	[128]
	In Vitro, wheat grain, ~115 µg/g	100	GC-MS and NMR analysis of degradation products (**3-oxo-DON (=3-keto-DON)** and **3-epi-DON**)	[128]
*Citrobacter freundii*	In Vitro, LB broth, 10 µg/mL	93.5	HPLC and UPLC-MS/MS analysis of DON degradation products (**3-keto-DON**, **DOM-1**)	[129]
*Pseudomonas* sp. Y1 + *Lysobacter* sp. S1	In Vitro, MSM medium, 50 µg/mL	100	HPLC for identification of degradation products (**3-epi-DON**)	[130]

Abbreviations: BHI, brain heart infusion; CMB, corn meal broth; MSM, mineral salts medium; PQQ, pyrroloquinoline quinone; MMYPF, minimum medium containing yeast extract, pyruvate, and fumarate; MMB2216, modified marine broth 2216 medium; LC-MS, liquid chromatography–mass spectrometry; RP-HPLC, reversed-phase high-performance liquid chromatography; LPA, lymphocyte proliferation assay; QToF LC-MS, quadrupole time-of-flight liquid chromatography–mass spectrometry; GC-MS, gas chromatography–mass spectrometry; UPLC-MS/MS, ultraperformance liquid chromatography-tandem mass spectrometry.

**Table 4:** Microbial species able to efficiently transform fumonisin B1 (FB1) into nontoxic compounds.

Species	Experimental Conditions and FB1 Concentration	FB1 Degradation Level, %	Approaches Used for Evaluation of Biotransformation Efficiency	Data From
*Sphingopyxis* MTA144	In Vitro, liquid medium	nd *	LC-MS analysis of FB1 metabolites	[133]
*Sphingomonas* sp. ATCC 5552	In Vitro, liquid medium, 0.5–1.0 mg/mL	nd	TLC and radiochemical analysis of FB1 metabolites	[139,140]
*Delftia/Comamonas* NCB 1492	In Vitro, liquid medium, 0.5 mg/mL	100	HPLC, GS-MS analysis of FB1 metabolites	[141]
*Serratia marcescens* 329-2	In Vitro, cell-free extract, 5 μg/mL	30.29	ELISA assay for residual FB1, MS-based proteomic analysis for enzyme expression	[142]
	In Vitro, cell-free extract, corn grain, 5 μg/mL	37	ELISA assay for residual FB1, MS-based proteomic analysis to evaluate enzyme expression	[142]
Bacterial consortium SAAS79	In Vitro, liquid medium, 50 μg/mL	~90	LS-MS/MS analysis of FB1 metabolites; MTT and MARC-145 bioassays to evaluate cytotoxicity	[143]
*Exophiala spinifera* 2141.10	In Vitro, MSM medium, 0.5–1 μg/mL	nd	NMR and mass spectroscopy analysis of FB1 metabolites	[135]
*Aspergillus welwitschiae* DAOMC 250207	nd	nd	LS-MS, NMR, isotope labeling to study FB1 metabolites; *Lemna minor* bioassay to evaluate toxicity	[136]

Abbreviations: MSM, Mineral Salts Medium; MTT, 3-(4,5-dimethylthiazol-2-yl)-2,5-diphenyltetrazolium bromide. * Not described.

## Data Availability

No new data were created or analyzed in this study. Data sharing is not applicable to this article.
